# Role of Nutritional Elements in Skin Homeostasis: A Review

**DOI:** 10.3390/biom15060808

**Published:** 2025-06-03

**Authors:** Nansong Jiang, Tao Quan, Ran Li, Yaoxing Chen, Ting Gao

**Affiliations:** 1 College of Veterinary Medicine, China Agricultural University, Beijing 100083, China; jiangnansong@faas.cn (N.J.); s20243051072@cau.edu.cn (T.Q.); 1214120476@ybu.edu.cn (R.L.); yxchen@cau.edu.cn (Y.C.); 2 Research Center for Poultry Diseases of Institute of Animal Husbandry and Veterinary Medicine, Fujian Academy of Agricultural Sciences, Fuzhou 350003, China; 3 Fujian Key Laboratory for Prevention and Control of Avian Diseases, Fuzhou 350003, China

**Keywords:** skin, aging, nutrient, antioxidant

## Abstract

Skin aging is the most prominent phenotype of human aging and is the result from the interaction of genetic and environmental factors. Improving skin aging is essential for maintaining the normal physiological function of the skin and the mental health of the human body. Existing studies have proposed many theories of aging, such as the free radical theory, mitochondrial theory of aging, etc., and accordingly have also proposed appropriate interventions. It is worth noting that many nutrients contained in the skin itself play an important role in maintaining skin health. During the aging process, a decrease in the levels of these nutrients is closely related to a weakening of the related functions. Therefore, supplementing these nutrients offers potential therapeutic options for improving skin aging. In this review, we summarize the important roles of nutrients in the skin and their potential in anti-aging, with the expectation of providing a therapeutic basis and clinical reference for delaying skin aging.

## 1. Introduction

Human skin is one of the largest organs of the body and plays many critical roles in maintaining body homeostasis, protecting the organism from external damage, maintaining body temperature and water balance, and transmitting sensory information, among other functions [1]. In order to be able to effectively fulfill these complex and important functions, the skin has developed the ability to provide a high degree of plasticity in response to changing environmental conditions while maintaining its structural integrity. It plays an important role in water and electrolyte homeostasis (epidermal barrier and sweat glands), thermoregulation (in vitro receptors), and immune response (skin-associated lymphoid tissue (SALT)). It is also an important sensory organ (with free nerve endings, Pacini vesicles, Meissner vesicles, Ruffini vesicles, Krause telomeres, and Merkel cells). It is also involved in metabolism and homeostasis in the body and is responsible for the removal, selective absorption, and storage of substances [2]. In addition, the skin is the body’s bearer of aesthetic functions, and its appearance, health, and well-being are inextricably linked to the body’s mental health [3].

The skin functions largely rely on the composition of nutrients in the host skin ecological environment. Recently, however, growing attention has been given to the effect of nutrition on skin homeostasis, with selected food components emerging as an alternative way to improve photoaging phenotypes [4]. Food and nutrition are associated with skin homeostasis in a two-way relationship. While nutrient deficiency has a vital effect on skin status, skin homeostasis also influences nutritional intake. Moreover, unhealthy diets result in a range of skin disorders, influence all the skin’s status from youth to aging, and induce injury to the skin, while a balanced diet is not only essential for the restoration of impaired skin but also for the skin’s phenotype and homeostasis [5,6]. Thus, the goal of the review is to research the different structures of the skin and their functions, the role played by nutrients contained in the skin itself versus nutrients from outside (fruits or vegetables) in the maintenance of skin health, and several measures that are effective in improving skin aging today. The views and data were collated from a range of literature databases and resources such as ScienceDirect, PubMed, Wiley, Springer, Taylor & Francis, INFLIBNET, Scopus, Google, and Google Scholar. The keywords and corresponding Relevant Medical Subject Headings (MeSH) terms used for searching databases include aging, impaired skin, nutrition, nutritious foods, balanced diet, unhealthy diet, fruits and vegetables, antioxidants, probiotics, and phytoestrogens. The selection process for relevant articles involved various titles obtained from the electronic database, then an evaluation of the abstracts of all analyzed and selected titles based on predetermined inclusion criteria.

## 2. Structures of the Skin

### 2.1. Epidermis

The epidermis is a highly cellular structure that is free of blood vessels and provides the body with a major barrier against environmental damage [7], protecting it from pathogens, ultraviolet radiation (UVR), and environmental pollution, while regulating the release of water from the body [8]. The epidermis is a continuously renewed structure that is mainly regenerated by epidermal stem cells. Cells located in the epidermis are divided into two main types: keratin-forming cells and non-keratin-forming cells, in which stem cells from the basement membrane differentiate to form keratin-forming cells, which are the main epidermal cell type forming the superficial layer of the epidermis. These cells are enriched in waveform proteins, junctional proteins, α-endoproteins, and nestin proteins, in addition to the family of keratins, which are alternatively and abundantly expressed in the different stages of keratin-forming cell differentiation. Non-keratin-forming cells consist of three main types of cells: melanocytes, Langerhans cells, and Merkel cells. Furthermore, the epidermis itself can be divided into five layers. From the outside to the inside, they are the stratum corneum, stratum pellucidum, stratum granulosum, stratum spinosum, and stratum basale.

### 2.2. Dermis

The dermis is the deeper area located below the epidermis and is the main component of the skin, providing the scaffolding and structure for the skin. The dermis is a dense and irregular layer of connective tissue, 2–3 mm thick, accounting for the majority of the skin’s thickness. It consists primarily of the fibroblast-produced extracellular matrix (ECM) that supports the skin’s vascular system, sensory nerve cells, and organelles such as sweat glands and hair follicles. Although these glands and organelles contain multiple cell populations, the dermal ECM itself consists primarily of sparsely distributed fibroblasts. These cells synthesize a complex structural matrix composed of collagen, elastin fibers, negatively charged hydrophilic proteoglycans, and hyaluronic acid. In addition to these major components, there are many less abundant ECM components, including soluble proteases, protease inhibitors, and cytokines. The dermis contains two main parts: the papillary layer and the reticular layer. The papillary dermis is the layer closest to the epidermis, directly beneath it, and has dermal papillae, while the reticular layer is located deep within the dermis and contains a large number of collagen fibers, elastic fibers, and glycosaminoglycans. Among them, hyaluronic acid has the ability to bind water and thus maintain skin moisture [9]. Fibroblasts are the most abundant cells in the dermis, and an important feature of these cells is their ability to synthesize and remodel the ECM, with remodeling supported by the synthesis of cleaved metalloproteinases and their inhibitors.

### 2.3. Epidermal-Dermal Junction

The epidermal-dermal junction is a basement membrane synthesized by basal keratinocytes and dermal fibroblasts and consists of a highly specialized extracellular matrix (composed of proteins such as laminin and type IV collagen) that mechanically anchors the two tissues while also playing a regulatory role in development and wound healing [10,11]. The DEJ is able to act as a mechanical support for dermal-epidermal adhesion and as a barrier to macromolecules, inflammatory cells, and tumor cells. In addition, it serves as an obligatory channel for nutrient delivery from the dermis to the epidermis, which can be accomplished to enable the penetration of nutrients and metabolic wastes, thus maintaining the overall homeostasis of the skin. Typically, in young, healthy human skin, the DEJ consists of embedded protrusions (papillae), similar to the villi of the small intestine, that maximize the surface area between the two layers, which improves the efficiency of nutrient delivery from the dermis to the epidermis [10]. On a mechanical level, this structure enhances adhesion between the dermis and epidermis and protects the dermis from physical trauma by diffusing external forces along the contact zone [12]. Furthermore, the absence of these papillae is a key marker of aging [13]. The structure of the epidermal-dermal junction is complex and consists of four different layers, namely the cytomembrane layer, the thylakoid layer, the dense layer, and the sub-dense layer region [10].

### 2.4. Subcutaneous Tissue

The subcutaneous tissue is a structure consisting of loose connective and adipose tissue located deep within the dermis [14], which connects the skin to the muscles. The subcutaneous tissue gives the skin some mobility, and the thickness of the subcutaneous tissue varies considerably depending on the individual, age, sex, nutrition, and disease; in general, it is thickest in the abdomen and buttocks and thinnest on the eyelids, dorsum of the hands, and dorsum of the feet. The subcutaneous tissue is rich in fat, which serves to cushion underlying tissues and organs from physical damage, provide insulation, and act as an energy reserve [15].

## 3. Nutrients Contained in the Different Structures of the Skin and Their Functions

### 3.1. Lipids

Skin lipids are mainly produced by sebocytes, keratin-forming cells, and the skin microbiome [16] (Table 1). Cortical cell-derived lipids are synthesized by sebaceous glands and secreted to the skin surface, with squalene, triglycerides (TG), fatty acids (FA), wax esters, cholesterol (CHOL), and cholesteryl esters as the main components. Lipid precursors are synthesized by keratin-forming cells and secreted in the form of lamellipodia, which ultimately release lipid precursors and lipid synthase into the extracellular space of the stratum corneum (SC). Lipid precursors are then catalyzed by lipid synthase to produce lipids, which mainly consist of ceramides, fatty acids, and cholesterol; additionally, the skin microbiome is an important source of skin lipids, that should not be ignored [17,18,19]. The skin is colonized by a wide range of microorganisms, most of which produce skin lipids, such as short-chain fatty acids (SCFA), during metabolic processes [20]. Lipids are important metabolites with many important cellular functions [21], including energy metabolism, signal transduction, enzyme activation, transmembrane transport, cell proliferation, development, differentiation, and apoptosis. In the classification system provided by Lipid MAPS, lipids can be categorized into eight groups, including fatty acids (FA), glycerol lipids (GL), glycerophospholipids (GP), sphingolipids (SP), sterol lipids (ST), isoprenoid lipids (PR), glycolipid lipids (GL), and polyketides (PK). Importantly, lipid composition provides a direct readout of the cellular metabolic state, and changes in lipid metabolism are important factors in the pathogenesis of respective diseases [22,23,24,25,26,27,28], such as metabolic disorders, several cancers, neurodegenerative diseases, and skin diseases.

In addition, skin condition is significantly influenced by skin lipids, and one of the earliest and widely studied aspects of skin lipid function is the maintenance of barrier function. The human skin is one of the largest organs that protects the body from irritants that affect human health, and skin lipids are among the most important compounds that maintain this barrier function; however, the influence of skin lipids on skin condition is not limited to this. They include (1) physicochemical functions, as they act as “mantle” that establishes the skin’s physicochemical barrier; (2) biochemical functions, as they signal in a complex network of signals originating at the epidermal level; and (3) microecological functions, as sebocytes and keratinocytes derive lipids that alter the composition of the microbial dermatoflora. Microbial metabolism produces lipids as signal initiators and signal transducers.

#### 3.1.1. Ceramides

It is always known that in addition to providing structural support to cell membranes, lipids produce cellular messengers (e.g., arachidonic acid, diacylglycerol, and ceramides) [29]. Ceramides belong to the sphingolipid class of compounds, and sphingolipids are structurally based on sphingomyelin or related bases. Ceramides are poorly soluble in water, but they are usually considered amphiphilic due to the presence of hydroxyl and amide bonds, which provide hydrophilicity to them.

Ceramides are mainly found in the epidermis, where they account for approximately 50% of the total epidermal lipid weight. Together with free fatty acids and cholesterol, ceramides constitute a hydrophilic extracellular lipid matrix, which is important for permeability barrier function [30]. In addition, ceramides act as active second messengers, regulate the proliferation and differentiation of keratinocytes, enhance the production of pro-inflammatory cytokines, and modulate the immune response. Epidermal ceramides are predominantly found in the stratum corneum and exhibit great molecular specificity. Several types of ceramides have been identified in the stratum corneum [31]. Normal-phase liquid chromatography-electrospray mass spectrometry analysis revealed more than 340 ceramides in the human stratum corneum [32]. The heterogeneity of the ceramide molecular structure allows for significant differences in the functions of different classes of ceramides, suggesting that they have different roles in skin homeostasis. Ceramides, fatty acids, and cholesterol constitute the extracellular lipid matrix of the epidermal barrier in approximately equimolar ratios and are assembled in dense orthogonal lateral stacks within the SC interstitium. Of these, ceramides are central to the structure and function of the permeability barrier. In certain skin diseases, such as atopic dermatitis and psoriasis, where skin barrier function is impaired, decreased ceramide levels are commonly observed. Reduced barrier function may be due to increased activity of enzymes such as ceramidase, sphingomyelin deacetylase, and glucosylceramide deacetylase, which enhance the degradation of ceramides and thus reduces the amount of ceramide in the skin. Patients with atopic dermatitis have lower concentrations of ceramide in the stratum corneum, which may be due to the increased activity of sphingosine deacetylase, an enzyme that degrades sphingolipids and produces sphingomyelin phosphorylcholine rather than ceramide [33]. As a signaling lipid that regulates epidermal function, ceramide activates several downstream signals, including ceramide-activated serine/threonine phosphatases (CAPS) such as protein phosphatase 1A [34], protein kinase C (PKC), histone D [35], and the kinase inhibitor RAS (KSR) [36]. Activation of these proteins induces a variety of responses, including cell proliferation, differentiation, and apoptosis in the epidermis [37,38]. Among these effects, ceramide-induced apoptosis, including apoptosis and autophagy, has been extensively studied and has attracted much attention.

#### 3.1.2. Squalenes

Squalene is secreted only by sebaceous glands and can be used as a marker to differentiate sebaceous gland lipids from stratum corneum lipids [39]. Squalene is an unsaturated triterpene consisting of six isoprenoids linked together, and due to its specific chemical structure, it possesses strong moisturizing and antioxidant properties. Analysis of sebaceous gland lipids by thin-layer chromatography (TLC) revealed that patients with atopic dermatitis (AD) had a twofold reduction in the amount of sebaceous gland lipids (especially squalene and wax esters) compared to healthy control subjects [40,41]. Additionally, the potential role of squalene and its peroxides in preventing sunburn and UV radiation has been reported [42]. Furthermore, waxes are more antioxidant than other lipids and may improve the water resistance of surfaces [43].

#### 3.1.3. Triglycerides

Triglycerides (TGs) are the most abundant components of sebaceous gland lipids. Li et al. showed that there is a correlation between the altered composition of TGs and the subtypes of AD [44]. A group of triglyceride levels (TG46:1, TG48:1, TG48:2, TG50:1, TG50:2, and TG50:3) was significantly lower in participants with AD and was not adjusted for age and sex. In contrast, TG46:2 and TG56:2 were significantly lower in AD participants after adjustment for age and gender [44].

#### 3.1.4. Free Fatty Acids

The ordered lipid accumulation in the stratum corneum is induced by free fatty acids (FFAs) with predominantly saturated chains with carbon chain lengths ranging from 12 to 30, with the majority of FFAs having chain lengths of 22, 24, or 26 carbon atoms [45,46]. Together with cholesterol sulfates, free fatty acids are the only lipids with charged/ionizable head groups in the stratum corneum [47], and thus may be necessary for the formation of the SC lipid bilayer. The presence of free fatty acids causes the SC epidermis to exhibit an acidic pH of approximately 4–5.5 [15].

#### 3.1.5. Cholesterol

Cholesterol is the most abundant lipid in the whole body and is part of the stratum corneum, plasma membrane, and intercellular lipid lamellae. Although basal cells can reabsorb cholesterol from the circulation, most of the cholesterol in the epidermis is synthesized in situ from acetate [48]. It is likely that the function of cholesterol in the epidermal barrier is to provide a degree of mobility and flexibility to the rigid and fragile membrane system.

**Table 1:** Functions performed by different lipids of the skin and their machine.

Items	Functionality	Lipids	Machine
Physicochemical functions	Barrier function	Squalene, wax esters, free fatty acids, cholesterol esters, cholesterol, phospholipids, triglycerides	Carbon chains form an epidermal permeability barrier [49,50]
	Protection from sunburn and UV radiation	Squalene, triglycerides	
	Waterproof	Wax ester	
	Antioxidant	Wax ester	
Biochemical function	Stimulates lipid synthesis	Unsaturated fatty acids, e.g., AA, LA	PPARγ activation [51,52]
	Promotes terminal sebocyte differentiation	Unsaturated fatty acids, e.g., AA, LA	PPARγ activation [51]
	Stimulates cell proliferation and migration	Ceramide-1-phosphate, Sphingosine-1-phosphate	Inhibition of TNFα activation and Toll-like receptor 4-induced NF-kB activation [53]
	Inhibits proliferation of keratinocytes	Unsaturated fatty acid	PPARγ activation [51]
	Apoptosis	Ceramide	Activation of TNF receptors
	Inhibition of apoptosis	Ceramide-1-phosphate; Sphingosine-1-phosphate	Inhibition of TNFα activation and Toll-like receptor 4-induced NF-kB activation [53,54]
	Inflammations	AA	Production of leukotriene B4 and prostaglandin E2
	Anti-inflammatory	Unsaturated fatty acid	PPARγ activation [51] and inhibition of Langerhans cell function; decreased expression of adhesion molecules
	Anti-aging	Unsaturated fatty acid	Inhibition of TNFα activation and Toll-like receptor 4-induced NF-kB activation [53,54]
Microecological function	Against some sensitive skin microbiomes such as Staphylococcus aureus	Short-chain fatty acid	Inhibition of histone deacetylase (HDAC) [55]
	Glucococcus antiglucosus	Sapienic acid	Membrane depolarization [56]
	Gram-positive bacteria	Palmitoleic acid	Membrane depolarization [56]
	Promotes differentiation of preadipocytes to adipocytes	Butyrate	Inhibition of histone deacetylase (HDAC) [55]

### 3.2. Proteins

The proteins in the skin are mainly composed of fibrous proteins. Fibrous proteins are primarly found in the dermis. The properties of fibers made up of different proteins vary and can be classified as follows:

#### 3.2.1. Collagen Fibers

Collagen fibers are the main component of dermal fibers, accounting for about 95%. Collagen fibers are tough and tensile, giving the skin tension and toughness against external mechanical damage while storing large amounts of water. Collagen fibers located in the superficial plexiform papillary layer are thin and have different directions, while collagen fibers in the deep reticular layer become thicker and intertwine parallel to the skin to form a network. Of these, collagen type I is the most common collagen, which accounts for approximately 50–80% of the dry weight of the entire dermal matrix [57]. It reduces skin aging caused by UV light and is present throughout the dermis in large bundles, larger in diameter than the embryonic-derived collagen type III. Type III collagen accounts for 10–15%. In addition to this, other important collagen types in the dermis include collagen types IV, VI, and VII. Type IV collagen is connected to laminin-6 and is found mainly in the basement membrane, whereas type V collagen is ubiquitous in connective tissues (about 10% of the dry weight of the dermis), where it establishes a separate network structure in the reticulation of type I and type III collagen [58]. Type VII collagen forms anchoring fibers at the dermal-epidermal junction, and additionally, its amount is significantly reduced in aging skin exposed to prolonged ultraviolet light [59]. X Type VII, called the motile version, is present at hemibridged granules; it connects to cenadherin-5, and it is also the main ligand for the integrins α-6-β-4. Type V collagen accounts for approximately 5% of the dermal matrix [60].

#### 3.2.2. Elastic Fibers

Elastin is a gelatinous protein and is the main component of elastic fibers. In addition, the elastin molecules constituting elastic fibers have the structural characteristic of curling; when subjected to external forces, the curled elastin molecules stretch and lengthen, and after removing the external forces, the elongated elastin molecules return to the curled state, as if they were springs. Therefore, elastic fibers are rich in elasticity but poor in toughness, and elastin accounts for only 2% of the total dry weight of the skin [61]. Elastic fibers are more than collagen fibers intertwined and twisted together. The direction of the papillary layer of elastic fibers is perpendicular to the epidermis so that the skin can be bounced back to its original position after being touched and pressed; the direction of the reticular layer of elastic fibers is the same as that of the collagen fibers and is parallel to the skin surface so that the collagen fibers can be restored to their original state after being pulled so that the skin has lateral elasticity and compliance, and it has a protective effect against external mechanical damage.

Elastin fibers exist in a different state compared to collagen, which exists only in a mature state throughout the dermis. Elastin is first synthesized as a water-soluble precursor substance and then cross-linked. The maturity of elastin increases through extensive cross-linking; that is, the molecular solubility decreases and then increases, with only mature elastic fibers present deep in the dermis, whereas immature fibers are still present in the superficial dermis, i.e., the papillary layer. The mature fibers consist of an irregular core of elastin as well as surrounding microprimary fibers that are rich in elastin (90%), and the most mature elastic fibers are found at the very bottom of the dermal reticular layer. They are all closely related to the elasticity of the skin.

Elastin is rich in glycine and proline, as is collagen, but they differ in that elastin contains only small amounts of hydroxyproline and is free of hydroxylysine [62]. Its amino acid composition is highly nonpolar. Prolonged exposure to ultraviolet light leads to the degradation of elastic fibers, and some irregularities can be clearly observed under the light microscope, a condition known as elastic tissue degeneration.

#### 3.2.3. Netted Fibers

They are more naïve fibrillar collagen fibers and are found in small numbers in the dermis. Reticular fibers are usually observed as a meshwork of fine protofibrils stained black by silver impregnation, which are usually located subcutaneously and cover the surface of muscle cells, adipocytes, etc. Under the electron microscope, the reticulated fibers are observed as individual collagen fibers or as small bundles of protofibrils, which are very thin in diameter, about 30 nm, and uniformly thick or thin. In silver-impregnated specimens, individual protofibrils in the reticular fibers were densely encapsulated by coarse metal particles, possibly be due to the high content of glycoproteins surrounding the protofibrils [63].

#### 3.2.4. Polyfilament Proteins

The physical barrier of the skin consists mainly of the SC, while the nucleated epidermis provides additional support. In particular, cell-cell junctions and associated cytoskeletal proteins provide other important components. Among these, keratins are arranged into highly ordered and condensed arrays through interactions with the matrix protein polyfilament proteins during the final stages of normal differentiation of keratin-forming cells. The role of polyfilament proteins in skin barrier homeostasis is only partially known. Polyfilament proteins play an important role in retaining moisture in the stratum corneum and are located in the granular keratin-forming cells and hypokeratinocytes of the stratum corneum [64]. Polyfilament proteins, together with keratin intermediates, aggregate during the terminal differentiation of the epidermis of malignant tumors and are an integral part of the envelope of the keratinized cells.

Polyfilament proteins aggregate keratin filaments into tight bundles, which promotes cell collapse into the flattened shape that characterizes keratinocytes in the keratinized layer [65]. Together, keratin and polyfilament proteins account for 80–90% of the protein mass of mammalian epidermis [66,67]. The importance of polyfilament proteins to epidermal barrier homeostasis has been shown in mouse models and human diseases, where this protein is aberrantly expressed in patients with common ichthyosis and atopic dermatitis [65,68,69]. Overexpression of filament polyprotein on mouse basal epidermis results in delayed barrier repair [70].

#### 3.2.5. Keratins

Keratins are the major structural proteins synthesized in keratin-forming cells; they assemble into a reticular pattern of intermediate filaments that extend from the perinuclear ring throughout the cytoplasm and terminate at the bridging and hemibridging granules. In keratin disorders, the network of filaments around the nucleus collapses, and thus interactions between neighboring cells are altered. In humans, genetic defects in suprabasal keratins result in hyperkeratosis and mild barrier defects manifested by a fragile and easily damaged epidermis. Keratin fundamentally affects epithelial cell structure (e.g., cell polarity and cell shape) and mitotic activity. The primary function of keratin and keratin filaments is to provide scaffolding for epithelial cells and tissues to maintain mechanical stress, preserve their structural integrity, and ensure mechanical elasticity [71].

### 3.3. Amino Acids

Each hundred grams of dried human skin tissue contains 71.9% collagen and 0.9% elastin. Skin collagen consists of glycine, proline, hydroxyproline, lysine, and hydroxylysine; elastin consists of glycine, alanine, valine, proline, and hydroxyproline. The properties and functions of each amino acid are described below in turn.

#### 3.3.1. Glycines (Gly)

Glycine is a major component of skin collagen, accounting for 1/3 of the amino acids in collagen and elastin and plays an important role in nutrition and metabolism. Glycine can be synthesized in the body and is a non-essential amino acid. However, increasing evidence suggests that the amount of glycine synthesized in vivo is insufficient to meet the metabolic needs of animals (including the synthesis of collagen, glutathione, and hemoglobin) [72]. Glycine has a facilitating effect on epidermal cell wandering and facilitates wound healing. Elastin fibers are essential extracellular matrix components of the skin that help maintain skin elasticity. During skin aging, elastin synthesis decreases, and activity is reduced, leading to skin laxity and loss of elasticity. Glycine induces the activation of elastin promoter activity and inhibits the degradation of elastin fibers by elastase in vitro [73].

#### 3.3.2. Prolines (Pro)

Proline, along with glycine, is the main amino acid involved in collagen synthesis, accounting for approximately 15% of the amino acid composition of collagen. It can be converted to hydroxylysine and hydroxyproline in the body to aid in the formation of collagen [74], Proline is beneficial for skin health and wound healing, stimulating cell migration and contributing to new tissue development, with blood proline levels near wounds being at least 50% higher than plasma levels during the early stages of wound healing [75]. Collagen supplements containing proline can improve skin barrier function, induce hyaluronic acid synthesis, and promote fibroblast growth and migration. In addition to its anti-aging and restorative effects, other functions of proline include support of the immune system, improvement of antioxidant status, improvement of intestinal health and nutrient absorption in the body, support of the metabolism, protection of the cardiovascular system, and participation in the skin’s metabolism, which is important for the overall health of the skin [76].

#### 3.3.3. Hydroxyprolines (Hpro)

Hydroxyproline is the main component of collagen, accounting for 14% of the total amino and imino content of collagen. Unlike glycine and proline, hydroxyproline is produced from proline-containing collagen. Specifically, hydroxyproline residues are formed by post-translational hydroxylation of proline in newly synthesized collagen [77]. Hydroxyproline is a structurally and physiologically important sub-amino acid in animals, which can be converted to glycine, enhancing the production of glutathione, DNA, hemoglobin, and proteins [78]. It has been shown that hydroxyproline can upregulate collagen biosynthesis and degradation, activate the collagen turnover cycle, and stimulate collagen metabolism [79].

#### 3.3.4. Lysines (Lys)

Lysine is one of the essential amino acids that can promote human development, enhance immune function, improve central nervous system function, act as an antiviral, promote fat oxidation, relieve anxiety, and more. At the same time, it can also promote the absorption of certain nutrients and can synergize with some nutrients to better facilitate the physiological functions of various nutrients. The recommended daily intake of lysine is 41 mg/kg body weight for adults and 89 mg/kg body weight for children. Lysine is involved in the synthesis of many proteins, such as skeletal muscle, enzymes, serum proteins, and peptide hormones. It is particularly important for the normal function of collagen and elastin. Lysine is not only found in the dermis, but some studies have found lysine in the epidermis as well [80]. Lysine plays an important role in the synthesis of fibronectin, collagen, and elastin [81].

#### 3.3.5. Cystines

Cystine is found in keratin in nails and hair and can be synthesized artificially, Cystine is a stable dimer consisting of two cysteine molecules linked by a disulfide bond and is obtained by food and food supplements. Purified cystine is 100% usable as a food supplement and is rapidly absorbed by the body and provides cells with enough cysteine for glutathione production [82]. Cystine assists in skin formation and is important for detoxification, protecting cells from copper toxicity by reducing the body’s ability to absorb copper. When metabolized, it releases sulfuric acid, which chemically interacts with other substances to increase the detoxification function of the entire metabolic system. In addition, it aids in the supply of insulin, which is necessary for the body to utilize sugar and starch. It also promotes cellular redox, enables vigorous liver function, promotes leukocyte proliferation, and prevents the development of pathogenic bacteria. It is used for hepatitis, alopecia areata, and leukopenia.

#### 3.3.6. Mercaptoethyl Amine

Cysteine is typically the limiting amino acid synthesized from glutathione, and increasing the supply of cysteine or its precursors enhances glutathione synthesis. Moreover, the main source of cysteine at the cellular level is cystine. While glutathione is a tripeptide composed of cysteine, glycine, and glutamic acid. It is a powerful intracellular antioxidant, and its antimelanogenic activity has been demonstrated [83,84,85].

#### 3.3.7. Arginines (Arg)

Arginine can be formed as an intermediate in the urea cycle in the mammalian liver, but there is no net synthesis of arginine in the liver [86]. There are no reports on the cutaneous urea cycle, and in humans, arginine is synthesized from glutamine, glutamate, and proline via the long renal axis. Arginine is a conditionally essential amino acid for children and adults [87]. It has the ability to repair damaged skin [88].

### 3.4. Vitamins

#### 3.4.1. Vitamin C

Normal skin contains high levels of vitamin C, comparable to other body tissues and much higher than plasma concentrations, indicating active circulatory accumulation, and most of the vitamin C in the skin is located in intracellular compartments at concentrations in the millimolar range [89,90]. It is transported from blood vessels in the dermis into the cells. Several reports have shown low levels of vitamin C in aging or photodamaged skin, and it has also been reported that overexposure to oxidative stress through pollutants or UV radiation is associated with the depletion of vitamin C levels in the epidermis [91,92]. In fact, there is more vitamin C in the epidermis than in the dermis, with a 2- to 5-fold difference between the two layers [90]. Levels of vitamin C in the skin are similar to those of other water-soluble antioxidants such as glutathione [90,92]. There are indications that vitamin C is present in the epidermal stratum corneum in a concentration gradient [93]. Vitamin C concentrations are lowest on the outer surface of the epidermis and increase dramatically in the deeper layers of the stratum corneum, reflecting exocytotic cellular depletion due to prolonged exposure to the environment [93].

Vitamin C is a cofactor for proline and lysine hydroxylases, stabilizes the tertiary structure of collagen molecules, and also promotes collagen gene expression [94,95], which protects against skin oxidation, contributes to the fight against skin aging, and plays a role in signaling pathways that play a role in cell growth and differentiation. Additionally. In vitro studies have shown that vitamin C can play a role in the differentiation of keratinocytes [96]. It promotes the synthesis of barrier lipids during differentiation and increases the formation of the keratinized envelope. There is evidence that vitamin C increases the proliferation and migration of dermal fibroblasts [97,98], functions that are essential for wound healing. Vitamin C deficiency can lead to significant skin problems; for example, early signs of scurvy include fragile skin and poor wound healing [99,100]. In contrast, signs of aging in human skin can be improved by providing vitamin C, which is supported by many studies [94,101].

#### 3.4.2. Vitamins D

Vitamin D is a fat-soluble vitamin that is not only a nutrient required for bone health but also an essential nutrient needed to regulate many physiological functions [102]. Vitamin D can be obtained from the diet or its cholesterol precursor in the skin through the effects of UV exposure. This biologically active form requires two subsequent hydroxylation reactions to obtain active hormonal activity. Vitamin D can also be synthesized in the skin, starting with the fact that the rate of vitamin D synthesis in the granular layer is regulated by the dual processes of pigmentation and keratinization of the overlying stratum corneum, which allow only a defined amount of solar UV irradiation to penetrate the outer layers of the skin and reach the areas where vitamin D is synthesized.

#### 3.4.3. Vitamins B

Thiamine (vitamin B1), riboflavin (vitamin B2), niacin (vitamin B3), pyridoxine (vitamin B6), and biotin (vitamin B7) are water-soluble vitamins that are essential for normal cellular metabolism and red blood cell synthesis [103,104,105]. These vitamins are stored in small quantities but play a role in the skin that cannot be replaced by other vitamins. The properties of the different B vitamins are described separately below.

##### Vitamin B3

Niacin (vitamin B3) is a water-soluble B vitamin that has a variety of physiological functions. It acts as a hydrogen electron donor and acceptor and is essential for adenosine triphosphate production, macronutrient metabolism, DNA repair, and cell signaling. Niacin deficiency can lead to dermatitis, diarrhea, and dementia, and the initial stages of deficiency are usually characterized by nonspecific symptoms such as lethargy, weakness, loss of appetite, mild digestive disturbances, and mental or emotional distress [106].

##### Vitamin B6

Vitamin B6, also known as pyridoxine, is a multifunctional coenzyme involved in many biochemical reactions, including amino acid metabolism, carbohydrate metabolism, and lipid metabolism. It is also effective in cognitive development for neurotransmitter synthesis, immune function for interleukin-2 production, hemoglobin formation, and gene expression [107].

##### Vitamin B7

Vitamin B7, also known as biotin, is an essential cofactor for mammalian carboxylase enzymes, which are involved in important metabolic pathways in humans. Biotin deficiency is rare in humans, but its deficiency leads to neuromuscular dysfunction, alopecia, and dermatitis in humans [108]. Additionally, despite the fact that there is no conclusive evidence to support that biotin can be used to treat skin disorders, many physicians frequently recommend biotin for the treatment of skin disorders, with 66% of dermatologists reporting that they recommend dietary supplements to their patients to improve their skin, hair, and nails [109].

##### Vitamin B12

Vitamin B12, also known as cobalamin, is a water-soluble vitamin that is important in hematology and the nervous system, and it has a complex relationship with the skin. Altered levels of cobalamin can lead to disease. The biosynthesis and metabolism of cobalamin are complex, and disease may be associated with alterations in this metabolic pathway. Skin manifestations of cobalamin deficiency include hyperpigmentation, changes in hair and nails, and changes in the oral cavity, including glossitis. In addition, several skin disorders, including vitiligo, stomatitis, atopic dermatitis, and acne, have been associated with cobalamin excess or deficiency [110,111].

#### 3.4.4. Vitamin E

Vitamin E is a group of lipophilic compounds comprising 76 meso2tocopherols (α-, β-, γ-, and δ-tocopherols) and 77 tocotrienols (α-, β-, γ-, and δ-tocotrienols) [112,113]. Vitamin E is synthesized exclusively by plants [113], and all forms are supplied to the body through food. α-Tocopherol is the most important form, as it shows an affinity for a specific protein, α-TTP, which binds and transports only this form of the vitamin, while the rest of the dietary forms are metabolized in the liver and excreted in the bile [114,115].

Vitamin E plays an important role in maintaining skin health by contributing to the skin’s antioxidant defenses and protecting the epidermis and dermis from oxidative stress induced by environmental factors. Vitamin E is the major fat-soluble antioxidant in humans [116]. Due to the antioxidant properties of vitamin E and its ability to scavenge free radicals and become part of the lipid structure, it prevents lipid peroxidation and slows down skin aging [115]. α tocopherol has been shown to reduce the amount of 8-hydroxydeoxyguanosine indirectly produced by reactive oxygen species, so it reduces the amount of ROS-induced DNA damage, thus helping to slow down the development of skin cancer. Several studies have also shown that vitamin E has strong photoprotective, firming, moisturizing, and anti-aging properties and improves the elasticity, structure, and suppleness of the epidermis and dermis. It is believed that vitamin E bound to intercellular and lipid structures protects the skin from solar UVB radiation, thereby preventing redness and swelling [116,117].

### 3.5. Minerals

**Figure 1:**
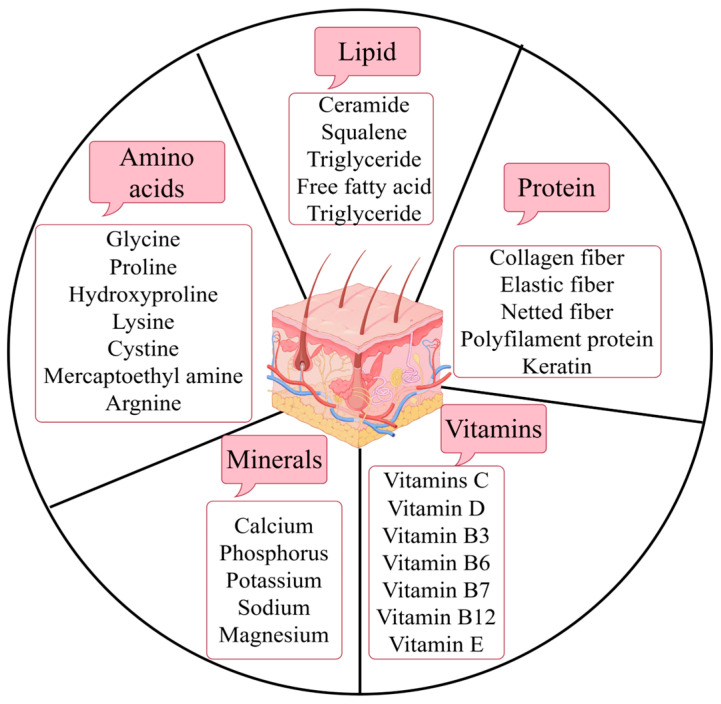
Nutrients contained in the different structures of the skin. The nutrients contained in the different structures and cells of the skin include lipids, proteins, amino acids, vitamins, and minerals.

Minerals, along with vitamins, are essential trace elements that cannot be synthesized by the body and therefore must be obtained through food. A healthy diet should ensure adequate intake of macronutrients (calcium, phosphorus, potassium, sodium, and magnesium) and trace minerals (iodine, sulfur, zinc, iron, cobalt, copper, manganese, and selenium). Minerals are responsible for the functions of the skeletal, circulatory, nervous, and endocrine systems. They also have many health benefits as cofactors and coenzymes in various enzyme systems and help regulate and harmonize biochemical and physiological functions. Micronutrient deficiencies can adversely affect human development and health, including the function and appearance of the skin [118]. In the context of skin aging, we also need to focus on the roles of minerals such as selenium, zinc, copper, and silicon (Figure 1).

## 4. Nutrients Contained in Fruits and Vegetables and Their Functions

As part of the daily diet, fruits and vegetables play a vital role in human health. They are rich in polyphenols, polysaccharides, dietary fiber, vitamins, minerals, natural pigments, and other active ingredients with antioxidant properties that protect the skin from free radical-induced cellular damage, maintain skin radiance, and prevent acne, pigmentation, and fine lines [119]. Consumption of fruits and vegetables also helps to reduce inflammation, brighten skin tone, and even lighten discoloration. Thus, bioactive compounds from a variety of plants are increasingly being used not only for body care, but also as ingredients that provide nutrients and influence the biological functions of the skin [120]. For example, polysaccharides are one of the most important active constituents in fruits and vegetables, with a large number of functional groups, high bioavailability, no toxic side effects, structural stability not easy to be denatured due to temperature and pH changes, as well as extraordinary bioactivities such as anti-aging, anti-inflammatory, antioxidant, antimicrobial, anticancer, digestive aid, immune modulation, anti-fatigue, lipid-lowering, and glucose-lowering [119]. The seeds of various fruits are also a source of oils, extracts, powders (flours), and a wide range of beneficial bioactive compounds [121]. Increased intake of green leafy vegetables and yellow vegetables was significantly associated with reduced wrinkles [122]. The Mediterranean diet, which is mainly characterized by a high intake of fruits, vegetables, legumes, nuts, and whole grains, is one of the most widely evaluated and described dietary patterns in the literature with proven health benefits [123]. The unique balanced composition of unsaturated fatty acids and vitamins in sea buckthorn oil has made it a popular ingredient in cosmetic and biomedical products, as it imparts a healthy and beautiful appearance to the skin and helps in caring for dry, rapidly aging skin, wounds, skin pigmentation, and infections [124].

### 4.1. Healthy Fats

Omega-3 and omega-6 polyunsaturated fatty acids are usually considered essential because humans cannot synthesize them on their own. Therefore, these fatty acids are obtained through various biochemical reactions in the diet [125]. Omega-3 polyunsaturated fatty acids significantly contribute to the production and circulating levels of specific anti-inflammatory and pro-resolving mediators i.e., autoreactive substances, including the D-series of antispasmodulins, protectins, and maresins derived from docosahexaenoic acid (DHA, C22:6 n-3), as well as the D-series from eicosapentaenoic acid (EPA, C20:5 n-3), an E-series antispasmodic [126]. Common plant sources of polyunsaturated fatty acids are vegetable oils such as corn oil, canola oil, flaxseed oil, safflower oil, soybean oil, and walnuts. DHA and EPA are omega-3 fatty acids found in fish and shellfish, whereas common plant sources of monounsaturated fatty acids are canola oil, safflower oil high in oleic acid, olive oil, sunflower oil, and nuts [127].

Omega-3 and omega-6 polyunsaturated fatty acids (PUFAs) are essential for maintaining of cellular function and are important components of cellular phospholipid membranes. Omega-3 polyunsaturated fatty acids and their metabolites also confer a variety of biological properties in the body, such as influencing neuronal function and reducing systemic inflammation. The commonly recommended optimal dietary ratio of omega-6 to omega-3 is 4 to 1, while higher ratios have been associated with autoimmune and cardiovascular diseases [128]. Anti-inflammatory PUFA-based supplementation and balanced nutrition are not only critical for maintaining and improving overall skin health, but also for the management of various skin disorders [129].

### 4.2. Carotenoids

Carotenoids are mainly tetraterpenoid compounds synthesized by plants, algae, fungi, and bacteria, with lipophilic pigments that are important for the diet, with greater variation and concentration of plant carotenoids. Fruits and vegetables rich in carotenoids have mainly antioxidant properties that help fight the incidence of certain diseases. The most common carotenoids that act in a dose- and time-dependent manner include α-carotene, β-carotene, β-cryptoxanthin, saffronin, curcumin, lycopene, lutein, and zeaxanthin [130]. α-carotene, β-carotene, and β-cryptoxanthin are known as vitamin A precursor carotenoids because they can be converted to retinol, whereas lutein, lycopene, and zeaxanthin are non-provitamin A precursor carotenoids with no vitamin A activity. However, β-carotene is the main and readily available carotenoid in the human diet and is found mainly in dark green, red, purple, yellow and orange fruits and vegetables, such as apricots, carrots, mangoes, papaya, spinach, pumpkin and sweet potatoes [131,132]. Immunomodulatory vitamin A plays an important role in the regulation, proliferation, and differentiation of a wide range of cells, including skin cells such as fibroblasts and keratin-forming cells, thereby stimulating the synthesis of collagen and elastin by fibroblasts, smoothing out deep wrinkles, and reversing photoaging [133]. Some of the roles of carotenoids include antioxidant, immune enhancement, inhibition of precancerous lesions, inhibition of mutation and transformation, quenching of non-photochemical fluorescence, reduction of the risk of certain cancers and cardiovascular events, and decreasing the risk of cataracts and macular degeneration by acting as pigments in the macula of primates [131].

## 5. Intervention of Different Nutrients in Skin Aging

### 5.1. Mechanisms of Skin Aging

Since birth, human beings are inevitably aging, and skin aging is a multifactorial process that can be categorized into endogenous aging (DNA damage, telomere shortening, mitochondrial impairment, etc.) and exogenous aging (ultraviolet rays, air pollution, smoking, etc.) according to the triggers. The former triggers aging mainly in non-sun-exposed areas of the skin, including the inner sides of the arms and buttocks, while the latter is mainly in skin that is frequently exposed to the sun, such as the face. Intrinsically aged skin appears thin on the outside and exhibits fine wrinkles, reduced elasticity, and marked dryness, often accompanied by itching. In contrast to these observations is extrinsically photoaged skin with epidermal thickening, dullness, and roughness. Capillary dilatation and pigment discoloration may also be observed in advanced and severe degrees of photoaging, which are the main skin aging-related changes found in Asian populations [134,135,136]. Skin aging is not only detrimental to physical health but also causes social impairment and can predispose to associated diseases, such as a significant increase in the incidence of dryness, itching, and skin irritation in older adults [137], alongside an increased risk of infections, chronic wounds (e.g., venous, pressure, or diabetic foot ulcers), as well as various types of dermatitis and malignant neoplasms (including melanoma) [138,139].

The aging process affects all skin layers and manifests itself in structural and functional changes [138]. The aged epidermis exhibits reduced barrier function and recovery after injury [140]. In addition, with age, the proliferative capacity of basal layer cells decreases, epidermal thickness decreases, and the contact surface area between the dermis and epidermis decreases, resulting in a smaller exchange surface for epidermal nutrient supply and a further diminution in the proliferative capacity of basal layer cells [141,142]. In addition to changes in the epidermis, the epidermal-dermal junction flattens, and the dermis thins. The flattening of the epidermal-dermal junction results in fewer cells, less nutrients, and reduced oxygen, leading to wrinkle formation. The dermal extracellular matrix (ECM) also exhibits structural and functional changes in intrinsically and extrinsically aged skin. These include changes in the accumulation of labile collagen and type III collagen, changes in the ratio of type I/III collagen [143], impaired synthesis of these ECM molecules [144], and changes in elastic fiber organization [145]. One study found that as the COLI/COLIII ratio increases, protofiber width and stiffness increase, while the affinity for cell-matrix binding decreases. Mixed matrices had the best activation of fibroblasts, with high cell polarization, production of rigid fiber bundles, high α-SMA expression, and higher levels of collagen synthesis. It is evident that the proportion of COLI and COLIII in the matrix is important, and together they determine the nature of the matrix and direct different cell biological responses [146]. Decrease in the number of fibroblasts also leads to alterations and degradation of the ECM, which is manifested by thinning of the skin, increased wrinkles, and loss of elasticity [147]. Aging also leads to anatomical changes affecting the skin’s microcirculation loop [112], which is characterized by a decrease in the size and number of blood vessels, associated with defective angiogenesis. The loss of dermal nutrient vascular density and exchange surface is accompanied by disorganization of the vascular network [148], which may also reveal changes in certain physiological features of aging skin, including pallor, decreased skin temperature, decreased UV-induced erythema, decreased nutrient intake, and decreased skin vascular reactivity [149]. These histologic changes are ultimately manifested as an overall thinning of the skin and diminished adipomuscular support, leading to skin laxity and increased wrinkles.

There are many mechanisms of skin aging, and researchers have proposed different models to explain the molecular basis of skin aging, including the theory of cellular senescence, the decline in cellular DNA repair and telomere loss, extranuclear mitochondrial DNA point mutations, oxidative stress, increased frequency of chromosomal anomalies, single-gene mutations, chronic inflammation, and others [13]. Some scientists have proposed that most of the effects are due to extrinsic factors and only 3% of aging is caused by intrinsic factors [102]. This paper focuses on the doctrine related to oxidative stress and telomere shortening.

It is believed that reactive oxygen species (ROS) play a key role in the dermal extracellular matrix alterations that are intrinsic to aging and photoaging. ROS can be produced by different sources, including the mitochondrial electron transport chain, peroxisomes, and endoplasmic reticulum (ER) localization proteins, as well as enzymes such as cyclooxygenases, lipoxygenases, xanthine oxidases, and nicotinamide adenine dinucleotide phosphate (NADPH) oxidases [101]. Under common conditions in the absence of ligands, cell surface receptor tyrosine kinase (RTK) activity is inhibited by the receptor protein tyrosine phosphatase RPTP, which dephosphorylates RTK. However, under UV radiation, cellular chromophores absorb energy and are excited to produce oxidation products and ROS. ROS inhibit RPTP activity by binding to cysteine at the catalytic site of RPTP [150], increasing the level of phosphorylated RTK and triggering downstream signaling pathways, including mitogen-activated protein kinase (MAPK) and subsequent nuclear factor-κB (NK-κB) and activation of transcription factor activator protein-1 (AP-1). Activated NF-κB and AP-1 inhibit collagen production and increase MMP gene transcription, leading to a decrease in collagen content in photoaged skin [151]. Notably, NF-κB has recently been found to be activated by the mammalian rapamycin complex 2/Akt/IκB kinase α pathway in aging and photoaging [152].

Telomere shortening is one of the earliest and most characteristic mechanisms that induce cellular senescence. Telomeres are located at the ends of linear chromosomes and consist of thousands of bases that contain tandemly repeated DNA sequences. Telomeres maintain the stability of chromosome structure by protecting chromosomes from degradation by nucleases and preventing end linkage between chromosomes [153]. When these telomeres remain intact, the life cycle of the cell is prolonged. However, with each cell division, the telomeres at the ends of the chromosomes are lost by about 30–200 base pairs (bp), and after multiple cell divisions the telomeres become very short, resulting in the loss of important DNA fragments during subsequent divisions, leading to loss of cellular function. The cell enters a state of value-added senescence, resulting in telomere loops or T-loops (TLoop) breaks and exposure of the 3′ end single-stranded overhangs. This exposure activates the tumor suppressor protein p53 and other DNA damage response proteins through interaction with the Werner protein, thereby inducing value-added cellular senescence or apoptosis, ultimately contributing to the onset of skin aging. It has also been noted that telomere loops can also naturally undergo disruption due to UV irradiation or other DNA damage [154].

Telomeres in skin cells may be particularly susceptible to accelerated shortening due to proliferation and the DNA-damaging agent ROS. Accelerated telomere loss after oxidative stress may be mediated by intracellular DNA repair processes, DNA damage accelerates telomere shortening in the skin, and telomeres also mediate melanin formation in senescent skin [155]. Keratin-forming cells are more susceptible to apoptosis and senescence than fibroblasts as a result of DNA damage induced by UV and ionizing radiation [4]. When keratinocytes are removed as apoptotic cells due to telomere shortening and DNA damage, fibroblasts may become senescent, affecting the epidermal growth process in the form of paracrine secretion and extracellular matrix deposition in the dermis.

Current anti-aging measures consist mainly of limiting the effects of senescence, particularly at the dermal and epidermal levels, and these approaches are diverse and involve a variety of strategies, including antioxidants, stem cell therapy, vitamin A-like acids, hormone replacement therapy, telomere modification, and dietary restrictions.

### 5.2. Antioxidants

Antioxidants act as reducing agents that can mitigate skin aging by neutralizing already formed ROS. ROS activates the MAPK pathway and subsequently increases MMP production, which degrades collagen. This can be prevented by antioxidants such as vitamin C and vitamin E or antioxidant enzymes such as superoxide dismutase, catalase, glutathione peroxidase, and coenzyme Q10 [156,157]. Some plants can also be used as natural sources of antioxidants, such as green tea and aloe vera [158]. It has been shown that a catechin (EGCG) in green tea prevents skin aging through the epidermal growth factor receptor (EGFR) pathway in a mouse model of aging, resulting in better skin structure than controls [159,160]. In addition, N-acetylcysteine, a form of the antioxidant glutathione, is capable of treating vascular and non-vascular neurological disorders as well as counteracting the age-related decline in tissue regeneration [161], which suggests its promising anti-aging applications in the skin.

However, it is worth noting that some researchers have suggested that antioxidant supplements have no preventive effect on chronic diseases and that excessive supplementation with β-carotene and vitamins A and E may be harmful and produce unwanted side effects [162,163]. Especially in well-nourished populations, the nearest source of antioxidants comes from our diet rather than from antioxidant supplements in pills or tablets [164], and a previous study found that EGCG induced significant death and DNA damage in normal cells of human lungs and skin through a reducing mechanism [165]. The aim of antioxidant therapy is to restore oxygen homeostasis rather than to completely eliminate all oxidants, as they have physiological functions [166]. Therefore, for the necessary clinical application of antioxidants, physicians should assess the patient’s status before prescribing them; complete inactivation of all ROS is not desirable, and antioxidant therapy appears to be beneficial in aging, including skin aging, only when ROS levels are reduced to those of healthy cells.

### 5.3. Stem Cell Therapy

Stem cell transplantation is a promising therapy for treating skin aging, and adipose tissue grafting improves the quality of skin at the subject site in addition to increasing skin volume [167]. Further experiments have shown that adipose-derived stem cells (ADSC) contribute to skin regeneration during aging [168,169]. In recent clinical trials, autologous fat grafting rejuvenated aging skin and increased periocular and perioral skin volume in subjects with an average age of 50 years [170,171]. The data show that ADSCs produce a range of growth factors, including vascular endothelial growth factor (VEGF), basic fibroblast growth factor (bFGF), transforming growth factor (TGF)-β1, TGF-β2, hepatocyte growth factor (HGF), keratinocyte growth factor (KGF), platelet-derived growth factor AA (PDGF-AA), and placental growth factor (PGF) [172]. ADSCs may affect the surrounding skin cells through these secretions, which reveals the mechanism by which fat grafting rejuvenates the overlying skin.

### 5.4. Retinoids

Vitamin A analogs, including retinol, retinaldehyde, retinoic acid, and synthetic analogs (tazarotene, etc.). Vitamin A analogs are chemically similar to vitamin A, including retinoids, etc. Retinoids are the first retinoids approved for clinical use, and the topical application of retinoids inhibits AP-1, which inhibits MMP expression and prevents collagen degradation [173]. Increases in epidermal thickness and anchoring protofibers observed, and intrinsically aged skin may also benefit from topical application of vitamin A analogs [174]. Additionally, 0.02% retinoic acid cream is now FDA-approved for the treatment of photoaging. Griffiths and colleagues reported a 48-week controlled trial comparing the clinical efficacy and tolerability of 0.025% and 0.1% retinoic acid creams. Both 0.025% and 0.1% retinoic acid significantly improved photoaging compared to control but there was no significant difference between the two concentrations of retinoic acid. However, 0.025% retinoic acid had fewer side effects. Moreover, Kligman conducted a 9 month experiment in which a 0.025% retinoic acid cream was applied once a day to the skin of the inner thighs of 6 women (average age 74). The results of the experiment demonstrated a significant improvement in skin condition, so that retinoic acid can combat natural aging in addition to photoaging. For significant skin improvement, retinoic acid was used for at least 6 months. No significant changes in dermal papillae were observed in the retinoic acid-treated group (whites) at 6 months, and new collagen formation was observed after 12 months. However, the experimental sample is small, and further validation is awaited.

### 5.5. Hormone Replacement Therapy

Hormone replacement therapy (HRT) is a medical treatment method that is often used for hormone replacement when specific hormones are missing from a patient’s body. This method involves: replacing the missing hormone with an agent containing the missing hormone that is injected into the patient’s body through an IV. In addition to being used to treat symptoms caused by menopause, HRT is also used to slows down the aging process of the skin; it improves skin thickness, collagen content and elasticity, and enhances hydration. However, some studies have shown that HRT increases the risk of breast cancer [175].

Skin aging is accelerated during menopause; however, many women and healthcare professionals are insufficiently informed on the impact of menopause on skin [176]. Declining estrogen detrimentally affects the skin’s ECM, which provides strength, elasticity, and resilience [177]. The relationship between dermal structural changes and altered skin function has been demonstrated by measuring the biomechanical properties of young, aged, and photoaged skin in parallel with histological analyses [178]. Estrogen levels also influence skin hydration, vascularity, and pigmentation [179], and women report a greater incidence of skin dryness and sensitivity in relation to the menstrual cycle and menopause [180,181]. However, the specific biological mechanisms underlying these observations are poorly understood, as are the respective contributions of epidermal and dermal changes to skin biomechanics. This indicates that epidermal lipid synthesis is altered postmenopause and is susceptible to regulation by HRT. In summary, increased viscoelastic skin distension post-menopause is reversible by HRT and may result from altered epidermal homeostasis and dermal morphology. Smaller R6 values are associated with improved skin condition, which is evident where decreased R6 is measured following the application of anti-aging skin care routines. Skewed CD44 expression and ceramide synthesis may contribute to declining epidermal barrier function and could represent key targets, beyond ECM rejuvenation, for improving skin function and appearance in midlife.

### 5.6. Telomere Modification

Cutaneous aging represents a multifaceted biological phenomenon that is influenced by an interplay of genetic predisposition, environmental factors, and lifestyle choices [182]. The length and integrity of telomeres exert a significant impact on the skin aging process, constituting one of the numerous influential factors [183], which has emerged as a prominent subject in the realm of anti-aging research and skin health. Although telomerase activation appears to be ideal for reversing skin aging, it is true that high levels of telomerase reverse transcriptase (TERT) expression in skin fibroblasts and keratinocytes lead to a significant enhancement of proliferative capacity [184] but at the same time an increased risk of epidermal carcinogenesis [185]. Therefore, further studies are needed to assess the safety of telomere lengthening.

Telomeres are specialized structures located at the ends of eukaryotic chromosomes, composed of repetitive DNA sequences that serve to safeguard against chromosomal injury and fusion [186]. Yet as cells undergo successive divisions, telomeres gradually shorten until they reach a critical length, thereby causing cell cycle arrest referred to as replicative senescence [187]. Telomere shortening is widely regarded as a priority indicator of cellular aging and closely related to the onset and progression of many age-related disorders. Recently, an increasing body of evidence has suggested a direct connection between telomere dynamics and skin aging, indicating that telomere length could be regarded as a valuable predictor for the process of skin aging and evaluation of anti-aging interventions [188,189]. The latest data advancements have suggested that telomere shortening is intricately associated not only with the conventional cellular aging process but also with biological pathways such as epigenetics, cell metabolism, and inflammatory response. For instance, it has been discovered that telomere shortening can impact gene expression patterns, thereby regulating cellular metabolism and inflammatory pathways [190], these alterations exert a vital effect on the process of skin aging. In addition, telomerase, an enzyme renowned for its ability to elongate telomeres, has garnered significant attention due to its regulation of activity and gene expression in retarding skin aging [191].

### 5.7. Dietary Interventions

Since it is still not technically feasible to reverse glycated proteins to their original state, the main strategy at present remains on the prevention of protein glycosylation. The problem, however, is that the diet provides not only sugars such as glucose and fructose, but also pre-formed angiogenic growth factors (AGFs), the latter of which are high in grilled, fried, and baked foods, but very low in foods prepared by water-based cooking such as poaching and steaming [192]. Thus, low-glycemic foods prepared with water reduce the intake of pre-formed exogenous AGFs and the endogenous production of physiologically glycated proteins. In the future, the search for drugs with deglycosylation ability will be a breakthrough discovery.

Some scientists believe that certain culinary herbs and spices, such as cinnamon, cloves, and oregano, inhibit fructose-induced glycosylation [193], and a number of compounds, including ginger, inositol, α-lipoic acid, carnitine, taurine, carnosine, flavonoids (e.g., green tea catechins), ammonium benzyl phosphate, α-tocopherol, niacinamide, pyridoxal, sodium selenite, riboflavin, zinc, and manganese, are also involved in inhibiting AGE formation [194,195]. More studies are needed to further validate these findings and reveal their inhibitory mechanisms.

In contrast, it is widely recognized that the nutritional status of macronutrients and micronutrients is important for skin health and appearance. Skin aging can be more effectively mitigated by supplementing nutrients that the skin synthesizes itself. Evidence for this is provided by the many vitamin-deficiency disorders that lead to serious skin diseases [196]. For example, dermatologic signs of B vitamin deficiency include blotchy red rashes, seborrheic dermatitis, and fungal skin and nail infections. Vitamin C deficiency scurvy is characterized by fragile skin, bleeding gums, and impaired wound healing [197,198].

### 5.8. Nutritional Interventions in Skin Aging

Nutritional status is critical for maintaining normal skin function during collagen synthesis and keratinocyte differentiation [118]. In addition, many of the body’s antioxidant components, such as vitamins C and E and selenium, are obtained from the diet, and these components are important for protection against UV-induced damage [199,200,201].

The epidermis, on the other hand, is a challenging environment for nutrient delivery because of its lack of vasculature to deliver nutrients to cells. Epidermal nutrient delivery depends on diffusion from the vascularized dermis [202]. Whereas diffusion of nutrients into the outermost layers of the epidermis is limited, in addition, there is also little movement of extracellular fluid between cells due to the complex lipid/protein cross-linking structure that forms the skin barrier, all of which makes it difficult for nutrients to reach the cells in the outermost layers of the epidermis, resulting in these cells to receive little nutritional support.

The skin can target nutrient delivery by topical application. However, because the stratum corneum acts as a barrier that prevents the passage of many substances, it is unlikely that nutrients delivered by topical application will readily penetrate deeper into the dermis, although some uncharged, lipid-soluble molecules can pass through the surface layer. Therefore, the function of the dermis is best supported by nutrients delivered through the bloodstream [203].

Many researchers have also emphasized the relationship between a properly balanced diet and the condition of the body, including the appearance and function of the skin [204]. The intake of essential nutrients in the daily diet is extremely important for the biological processes in young and aging skin [205]. Skin is a tissue with high proliferative potential, which explains why adequate intake of proteins, carbohydrates, and fats that contribute to cell production is so important [205,206,207]. The overall condition of the skin—including surface texture, color, and physiological properties—is a result of factors such as hydration, i.e., the presence of sufficient water in the stratum corneum, sebum content, and surface acidity. Natural moisturizing factors (NMFs), composed mainly of amino acids, play an important role in hydration and acidity [208]. Specific fatty acids are also important for maintaining the function of the skin barrier and the integrity of the stratum corneum [209]. A growing body of research suggests that a well-balanced diet significantly influences the skin aging process. Functional anti-aging components in foods include substances involved in the synthesis and metabolism of skin components (e.g., protein peptides and essential fatty acids), as well as substances that inhibit the degradation of skin components and maintain their structural integrity (e.g., substances that regulate the expression of enzymes, such as matrix metalloproteinases (MMPs) and activated protein 1 (AP-1)) [205]. Due to their antimutagenic, antioxidant, and free radical-scavenging properties, which protect the skin from UV-induced deleterious effects, a number of dietary plants may be useful supplements for skin care [210,211]. These include carotenoids and polyphenols such as quercetin, curcumin, apigenin, proanthocyanidins, and resveratrol. Other key elements of an anti-aging diet are vitamins and minerals with antioxidant properties [212].

**Figure 2:**
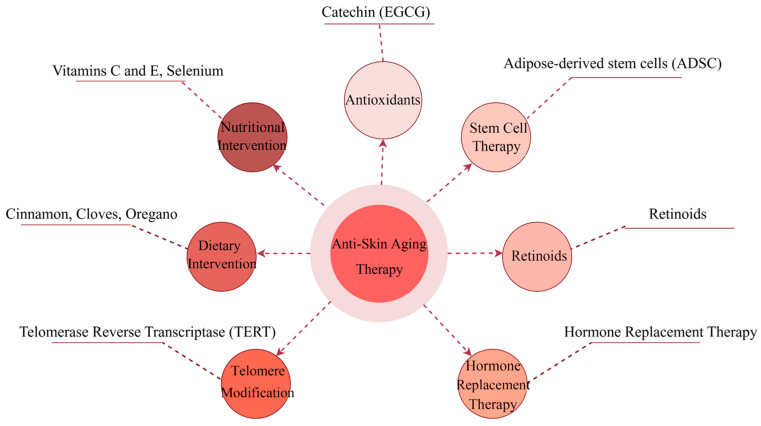
Several interventions against skin aging. These include antioxidant stem cell therapy, vitamin-likes treatments, hormone replacement therapy, telomere modification, dietary interventions, and nutrient interventions.

Substances that protect the skin from oxidative and UV damage, dehydration, and loss of elasticity include vitamins A, C, and E; selenium, zinc, copper, silica, and polyphenols; carotenoids; and essential polyunsaturated omega-3 and omega-6 fatty acids. A growing body of research suggests that a well-balanced diet significantly affects the skin aging process. These substances protect and restore the epidermal barrier, ensure proper levels of skin hydration, and protect against damage caused by external factors and inflammation. All of these nutrients have an intervening effect on skin aging (Figure 2).

## 6. Pharmaceutical or Cosmetic Topical in Skin Aging

Various offerings of plant origin have been observed as anti-aging offerings regulating one or more mediators associated with skin elasticity and wrinkle production. Over the past few years, potential anti-aging isolated compounds from plants were reported to increase skin elasticity by various pharmacological actions. Some xanthones from *Garcinia mangostana*, mainly α-mangostin, decreased UVB-caused skin wrinkles and suppressed epidermal thickening in hairless mice, in addition to other pharmacological influences [213]. Phenol compounds from *Dendrobium loddigesii*, including batatasin III, up-regulated collagen synthesis [214], whereas various limonoids from *Carapa guainensis*, mainly genudin, acetoxygenudin, andirolide H, hydroxygenudin methyl angolensate, and carapanoside C, improved collagen synthesis without cytotoxicity [215]. Triterpenoids from *Eriobotrya japonica*, mainly ursolic, pomolic, colosolic, and methyl colosolic acids, exhibited anti-aging activity by stimulating collagen and hyaluronic acid synthesis [216]. A plant hormone derived from the jasmonate-caused expression of proteoglycan in the human reconstituted epidermis [217], and a by-product polysaccharide obtained from red ginseng (*Panax ginseng*) inhibited UV-induced MMP-1 through activator protein-1 (AP-1) [218]. Finally, ferulic acid improved skin elasticity with significant bleaching, anti-redness, smoothing, and moisturizing activity in a clinical study [219].

On the other hand, plant-based extracts or differential herbal formulations have also been exhaustively studied in several in vitro models, with different pharmacological actions on several mediators of skin elasticity, according to the following classification:

1. Elastase suppress activity was determined after the use of the plant extracts prepared with *Eugenia dysenterica* [219], *Gastrodia elata* [220], *Litchi sinensis* [221], *Magnolia officinalis* [222], *Malaxis acuminata* [223], *Manilkara zapota* [224], *Nephelium lappaceum* [221], *Phyllanthus emblica* [224], *Sclerocarya birrea* [225], *Sylibum marianum* [224], *Spatholobus suberectus* [226], *Tamarindus indica* [221], and a polyherbal formulation containing *Nyctanthes arbor-tristis* leaf, unripe and ripe *Aegle marmelos* fruit pulp, and the terminal meristem of *Musa paradisiaca* extracts [227];
2. An up-regulation in elastin synthesis or gene expression was reported after treatments with plant extracts of *Piper cambodianum* [228] and *Bidens pilosa* [229];
3. An up-regulation in pro-collagen expression or synthesis was reported after treatments with plant extracts prepared from *Alchemilla mollis* [230], *Azadirachta indica* [231], *Camellia sinensis* [232], *Citrus junus* [233], *Trapa japonica* [234], and a mixture of plant extracts of *Kochia scoparia* and *Rosa multiflora* [235];
4. An up-regulation in collagen synthesis or expression was reported after treatments with plant extracts of *Andrographis paniculata* [236], *Cassia fistula* [237], *Camelia sinensis* [238], *Passiflora tarminiana* [239], *Physalis peruviana* [240], *Piper cambodianum* [228], *Solanum tuberosum* [241], and *Bidens pilosa* [229];
5. The regulation of hyaluronic acid synthesis was reported after treatments with plant extracts of *Cassia fistula* [237], *Penthorium chinense* [241], and *Salvia officinalis* [242];
6. The reduction of MMP-1 level was observed after treatments with plant extracts of *Alchemilla mollis* [230], *Allium sativum* [243], *Azadirachta indica* [231], *Camellia sinensis* [232], *Gastrodia elata* [221], *Kochia scoparia* [235], *Magnolia officinalis* [223], *Passiflora tarminiana* [239], *Penthorium chinense* [242], *Rosa multiflora* [235], and *Syzygium aromaticum* [244];
7. The reduction of MMP-2 level was observed after treatments with plant extracts of *Cassia fistula* [237], *Magnolia officinalis* [223], and *Pourthiaea villosa* [245];
8. A decrease in other metalloproteinases was reported after treatments with plant extracts of *Penthorium chinense* [241], *Piper cambodianum* [228], *Pousthiaea villosa* [245] and *Syzygium aromaticum* [244];
9. The suppression of the unspecific collagenase activity appeared after treatments with plant extracts of *Cassia fistula* [237], *Curcuma heyneana* [246], *Eugenia dysenterica* [220], *Hibiscus sabdariffa* [247], *Litchi chinens* [232], *Magnolia officinalis* [223], *Malaxis acuminate* [224], *Manilkara zapota* [225], *Nephelium lappaceum* [222], *Passiflora tarminiana* [240], *Phyllanthus emblica* [226], *Piper cambodianum* [228], *Sclerocarya birrea* [226], *Sylibum marianum* [225], and *Tamarindus indica* [222].

In addition, two clinical experiments were performed to research herbal formulas on skin elasticity. The research explored the anti-aging potential in mammalian skin of topical therapies containing a mixture of *Camellia sinensis* leaf, *Polygonum cuspidatum* root, *Ginkgo biloba* leaf, *Cynara scolymus* leaf, and *Selaginella tamariscina* and reported helpful influences on facial skin as well as an improvement in ECM protein production, although the reports are not statistically obvious [248]. An herbal preparation containing *Vaccinum vitis-idaea* and *Phyllanthus emblica* was orally administered to volunteers in a double-blind, placebo-controlled clinical experiment, generating obvious helpful influences on skin elasticity [249]. The effects were associated with improvements in skin thickness and stratum corneum water content and a decrease in facial wrinkles in a dose-dependent manner [249]. Furthermore, a phytocosmetic formulation containing *Thymus vulgaris*, after its topical application in volunteers, produced a decrease in perioral and crow’s feet wrinkles, accompanied by a decrease in nasolabial and smile lines, and an improvement in face oval remodeling [250].

## 7. Conclusions and Future Prospects

Skin aging is the direct manifestation of human aging, which is the result of the interaction of genetic and environmental factors. The improvement of skin aging is imperative and important to maintain normal physiological functions of the skin and people’s psychological health. In this paper, we review the various nutrients contained in the skin and their important role in maintaining skin health. Furthermore, we also analyze the mechanisms of skin aging as well as current interventions to slow down skin aging, which seems to be a promising approach by supplementing the nutrients contained in the skin itself. However, basic and clinical research to verify whether these additives can be applied in skin anti-aging is still very limited and deserves further exploration.

## Data Availability

Data will be made available on request.

## Abbreviations

The following abbreviations are used in this manuscript:

SALT	Skin-associated lymphoid tissue
UVR	Ultraviolet radiation
ECM	Extracellular matrix
TG	Triglycerides
FA	Fatty acids
CHOL	Cholesterol
SC	Stratum corneum
SCFA	Short-chain fatty acids
GL	Glycerol lipids
GP	Glycerophospholipids
SP	Sphingolipids
ST	Sterol lipids
PR	Isoprenoid lipids
SL	Glycolipid lipids
PKC	Protein kinase C
KSR	Kinase inhibitor RAS
TLC	Thin-layer chromatography
AD	Atopic dermatitis
TGs	Triglycerides
FFAs	Free fatty acids
HDAC	Histone deacetylase
Gly	Glycine
Pro	Proline
Hpro	Hydroxyproline
Lys	Lysine
Arg	Arginine
ROS	Reactive oxygen species
ER	Endoplasmic reticulum
NADPH	Nicotinamide adenine dinucleotide phosphate
RTK	Receptor tyrosine kinase
MAPK	Mitogen-activated protein kinase
NK-kB	Nuclear factor-kB
AP-1	Activator protein-1
EGFR	Epidermal growth factor receptor
ADSC	Adipose-derived stem cells
VEGF	Vascular endothelial growth factor
TGF	Transforming growth factor
HGF	Hepatocyte growth factor
KGF	Keratinocyte growth factor
PDGF-AA	Platelet-derived growth factor AA
PGF	Placental growth factor
HRT	Hormone replacement therapy
TERT	Telomerase reverse transcriptase
NMFs	Natural moisturizing factors
MMPs	Matrix metalloproteinases
AP-1	Activated protein 1

## References

[1] Lephart E.D. (2016). Skin aging and oxidative stress: Equol’s anti-aging effects via biochemical and molecular mechanisms. Ageing Res. Rev..

[2] Ndiaye M.A., Nihal M., Wood G.S., Ahmad N. (2014). Skin, reactive oxygen species, and circadian clocks. Antioxid. Redox Signal..

[3] Montagna W., Carlisle K. (1979). Structural changes in aging human skin. J. Investig. Dermatol..

[4] Geng R., Kang S.G., Huang K., Tong T. (2021). Boosting the photoaged skin: The potential role of dietary components. Nutrients.

[5] Greenberg S.A. (2020). Diet and skin: A primer. Cutis..

[6] Zohoori F.V. (2020). Chapter 1: Nutrition and Diet. Monogr. Oral Sci..

[7] D’Errico M., Lemma T., Calcagnile A., Proietti De Santis L., Dogliotti E. (2007). Cell type and DNA damage specific response of human skin cells to environmental agents. Mutat. Res..

[8] Fluhr J.W., Feingold K.R., Elias P.M. (2006). Transepidermal water loss reflects permeability barrier status: Validation in human and rodent in vivo and ex vivo models. Exp. Dermatol..

[9] Ni C., Zhang Z., Wang Y., Zhang Z., Guo X., Lv H. (2023). Hyaluronic acid and HA-modified cationic liposomes for promoting skin penetration and retention. J Control Release..

[10] Waller J.M., Maibach H.I. (2006). Age and skin structure and function, a quantitative approach (II): Protein, glycosaminoglycan, water, and lipid content and structure. Ski. Res. Technol..

[11] Xiong X., Wu T., He S. (2013). Physical forces make rete ridges in oral mucosa. Med. Hypotheses.

[12] Naylor E.C., Watson R.E., Sherratt M.J. (2011). Molecular aspects of skin ageing. Maturitas.

[13] Sharma A., Kuhad A., Bhandari R. (2022). Novel nanotechnological approaches for treatment of skin-aging. J. Tissue Viability.

[14] Baroni A., Buommino E., De Gregorio V., Ruocco E., Ruocco V., Wolf R. (2012). Structure and function of the epidermis related to barrier properties. Clin. Dermatol..

[15] Cui L., Jia Y., Cheng Z.W., Gao Y., Zhang G.L., Li J.Y., He C.F. (2016). Advancements in the maintenance of skin barrier/skin lipid composition and the involvement of metabolic enzymes. J. Cosmet. Dermatol..

[16] Grice E.A., Kong H.H., Conlan S., Deming C.B., Davis J., Young A.C. (2009). NISC Comparative Sequencing Program; Bouffard GG, Blakesley RW, Murray PR, Green ED, Turner ML, Segre JA. Topographical and temporal diversity of the human skin microbiome. Science.

[17] Lai Y., Di Nardo A., Nakatsuji T., Leichtle A., Yang Y., Cogen A.L., Wu Z.R., Hooper L.V., Schmidt R.R., von Aulock S. (2009). Commensal bacteria regulate Toll-like receptor 3-dependent inflammation after skin injury. Nat. Med..

[18] Naik S., Bouladoux N., Wilhelm C., Molloy M.J., Salcedo R., Kastenmuller W., Deming C., Quinones M., Koo L., Conlan S. (2012). Compartmentalized control of skin immunity by resident commensals. Science.

[19] Achermann Y., Goldstein E.J., Coenye T., Shirtliff M.E. (2014). Propionibacterium acnes: From commensal to opportunistic biofilm-associated implant pathogen. Clin. Microbiol. Rev..

[20] Han X., Gross R.W. (2022). The foundations and development of lipidomics. J. Lipid Res..

[21] Graessler J., Schwudke D., Schwarz P.E., Herzog R., Shevchenko A., Bornstein S.R. (2009). Top-down lipidomics reveals ether lipid deficiency in blood plasma of hypertensive patients. PLoS ONE.

[22] Meikle P.J., Christopher M.J. (2011). Lipidomics is providing new insight into the metabolic syndrome and its sequelae. Curr. Opin. Lipidol..

[23] Pietiläinen K.H., Róg T., Seppänen-Laakso T., Virtue S., Gopalacharyulu P., Tang J., Rodriguez-Cuenca S., Maciejewski A., Naukkarinen J., Ruskeepää A.L. (2011). Association of lipidome remodeling in the adipocyte membrane with acquired obesity in humans. PLoS Biol..

[24] Hu Q., Wang M., Cho M.S., Wang C., Nick A.M., Thiagarajan P., Aung F.M., Han X., Sood A.K., Afshar-Kharghan V. (2016). Lipid profile of platelets and platelet-derived microparticles in ovarian cancer. BBA Clin..

[25] Ahn B., Soundarapandian M.M., Sessions H., Peddibhotla S., Roth G.P., Li J.L., Sugarman E., Koo A., Malany S., Wang M. (2016). MondoA coordinately regulates skeletal myocyte lipid homeostasis and insulin signaling. J. Clin. Investig..

[26] Wang M., Han X. (2016). Advanced shotgun lipidomics for characterization of altered lipid patterns in neurodegenerative diseases and brain injury. Methods Mol. Biol..

[27] Bechmann L.P., Hannivoort R.A., Gerken G., Hotamisligil G.S., Trauner M., Canbay A. (2012). The interaction of hepatic lipid and glucose metabolism in liver diseases. J. Hepatol..

[28] Spiegel S., Foster D., Kolesnick R. (1996). Signal transduction through lipid second messengers. Curr. Opin. Cell Biol..

[29] Narangifard A., den Hollander L., Wennberg C.L., Lundborg M., Lindahl E., Iwai I., Han H., Masich S., Daneholt B., Norlén L. (2018). Human skin barrier formation takes place via a cubic to lamellar lipid phase transition as analyzed by cryo-electron microscopy and EM-simulation. Exp. Cell Res..

[30] Meckfessel M.H., Brandt S. (2014). The structure, function, and importance of ceramides in skin and their use as therapeutic agents in skin-care products. J. Am. Acad. Dermatol..

[31] YMasukawa Y., Narita H., Shimizu E., Kondo N., Sugai Y., Oba T., Homma R., Ishikawa J., Takagi Y., Kitahara T. (2008). Characterization of overall ceramide species in human stratum corneum. J. Lipid Res..

[32] Imokawa G.A. (2009). possible mechanism underlying the ceramide deficiency in atopic dermatitis: Expression of a deacylase enzyme that cleaves the N-acyl linkage of sphingomyelin and glucosylceramide. J. Dermatol. Sci..

[33] Chalfant C.E., Szulc Z., Roddy P., Bielawska A., Hannun Y.A. (2004). The structural requirements for ceramide activation of serine-threonine protein phosphatases. J. Lipid Res..

[34] Lozano J., Berra E., Municio M.M., Diaz-Meco M.T., Dominguez I., Sanz L., Moscat J. (1994). Protein kinase C zeta isoform is critical for kappa B-dependent promoter activation by sphingomyelinase. J. Biol. Chem..

[35] Heinrich M., Wickel M., Schneider-Brachert W., Sandberg C., Gahr J., Schwandner R., Weber T., Saftig P., Peters C., Brunner J. (1999). Cathepsin D targeted by acid sphingomyelinase-derived ceramide. EMBO J..

[36] Zhang Y., Yao B., Delikat S., Bayoumy S., Lin X.H., Basu S., McGinley M., Chan-Hui P.Y., Lichenstein H., Kolesnick R. (1997). Kinase suppressor of Ras is ceramide-activated protein kinase. Cell.

[37] Lee K.H., Zeng H. (2017). Aptamer-based ELISA assay for highly specific and sensitive detection of Zika NS1 protein. Anal. Chem..

[38] Smith K.R., Thiboutot D.M. (2008). Thematic review series: Skin lipids. Sebaceous gland lipids: Friend or foe?. J. Lipid Res..

[39] De Luca C., Valacchi G. (2010). Surface lipids as multifunctional mediators of skin responses to environmental stimuli. Mediat. Inflamm..

[40] Shi V.Y., Leo M., Hassoun L., Chahal D.S., Maibach H.I., Sivamani R.K. (2015). Role of sebaceous glands in inflammatory dermatoses. J. Am. Acad. Dermatol..

[41] Ohsawa K., Watanabe T., Matsukawa R., Yoshimura Y., Imaeda K. (1984). The possible role of squalene and its peroxide of the sebum in the occurrence of sunburn and protection from the damage caused by U.V. irradiation. J. Toxicol. Sci..

[42] Wertz P.W. (2009). Epidermal surface lipids. Dermatoendocrinol.

[43] Li S., Villarreal M., Stewart S., Choi J., Ganguli-Indra G., Babineau D.C., Philpot C., David G., Yoshida T., Boguniewicz M. (2017). Altered composition of epidermal lipids correlates with Staphylococcus aureus colonization status in atopic dermatitis. Br. J. Dermatol..

[44] Norlén L., Nicander I., Lundsjö A., Cronholm T., Forslind B. (1998). A new HPLC-based method for the quantitative analysis of inner stratum corneum lipids with special reference to the free fatty acid fraction. Arch. Dermatol. Res..

[45] van Smeden J., Boiten W.A., Hankemeier T., Rissmann R., Bouwstra J.A., Vreeken R.J. (2014). Combined LC/MS-platform for analysis of all major stratum corneum lipids, and the profiling of skin substitutes. Biochim. Biophys. Acta.

[46] Wertz P.W. (1992). Epidermal lipids. Semin. Dermatol..

[47] Karim N., Phinney B.S., Salemi M., Wu P.W., Naeem M., Rice R.H. (2019). Human stratum corneum proteomics reveals cross-linking of a broad spectrum of proteins in cornified envelopes. Exp. Dermatol..

[48] Chandrasekaran S.K., Shaw J.E. (1978). Factors influencing the percutaneous absorption of drugs. Curr. Probl. Dermatol..

[49] Chandrasekaran S.K., Bayne W., Shaw J.E. (1978). Pharmacokinetics of drug permeation through human skin. J. Pharm. Sci..

[50] Fluhr J.W., Man M.Q., Hachem J.P., Crumrine D., Mauro T.M., Elias P.M., Feingold K.R. (2009). Topical peroxisome proliferator activated receptor activators accelerate postnatal stratum corneum acidification. J. Investig. Dermatol..

[51] Tyagi S., Gupta P., Saini A.S., Kaushal C., Sharma S. (2011). The peroxisome proliferator-activated receptor: A family of nuclear receptors role in various diseases. J. Adv. Pharm. Technol. Res..

[52] Kim T.J., Kang Y.J., Lim Y., Lee H.W., Bae K., Lee Y.S., Yoo J.M., Yoo H.S., Yun Y.P. (2011). Ceramide 1-phosphate induces neointimal formation via cell proliferation and cell cycle progression upstream of ERK1/2 in vascular smooth muscle cells. Exp. Cell Res..

[53] Gangoiti P., Granado M.H., Arana L., Ouro A., Gomez-Muñoz A. (2010). Activation of protein kinase C-alpha is essential for stimulation of cell proliferation by ceramide 1-phosphate. FEBS Lett..

[54] Moissl-Eichinger C., Probst A.J., Birarda G., Auerbach A., Koskinen K., Wolf P., Holman H.N. (2017). Human age and skin physiology shape diversity and abundance of Archaea on skin. Sci. Rep..

[55] Fearon D.T., Locksley R.M. (1996). The instructive role of innate immunity in the acquired immune response. Science.

[56] Oikarinen A. (1990). The aging of skin: Chronoaging versus photoaging. Photodermatol. Photoimmunol. Photomed..

[57] Berardesca E. (2002). Disorders of skin barriers: Clinical implications. J. Eur. Acad. Dermatol. Venereol..

[58] Craven N.M., Watson R.E., Jones C.J., Shuttleworth C.A., Kielty C.M., Griffiths C.E. (1997). Clinical features of photodamaged human skin are associated with a reduction in collagen VII. Br. J. Dermatol..

[59] Glaser D.A. (2003). Cosmetic Dermatology: Principles and Practice: Leslie Baumann.

[60] Weinstein G.D., Boucek R.J. (1960). Collagen and elastin of human dermis. J. Investig. Dermatol..

[61] Mostafavi Yazdi S.J., Baqersad J. (2022). Mechanical modeling and characterization of human skin: A review. J. Biomech..

[62] Ushiki T. (2002). Collagen fibers, reticular fibers and elastic fibers. A comprehensive understanding from a morphological viewpoint. Arch. Histol. Cytol..

[63] Hsu C.Y., Henry J., Raymond A.A., Méchin M.C., Pendaries V., Nassar D., Hansmann B., Balica S., Burlet-Schiltz O., Schmitt A.M. (2011). Deimination of human filaggrin-2 promotes its proteolysis by calpain 1. J. Biol. Chem..

[64] Palmer C.N., Irvine A.D., Terron-Kwiatkowski A., Zhao Y., Liao H., Lee S.P., Goudie D.R., Sandilands A., Campbell L.E., Smith F.J. (2006). Common loss-of-function variants of the epidermal barrier protein filaggrin are a major predisposing factor for atopic dermatitis. Nat. Genet..

[65] Roop D. (1995). Defects in the barrier. Science.

[66] Nemes Z., Steinert P.M. (1999). Bricks and mortar of the epidermal barrier. Exp. Mol. Med..

[67] Smith F.J., Irvine A.D., Terron-Kwiatkowski A., Sandilands A., Campbell L.E., Zhao Y., Liao H., Evans A.T., Goudie D.R., Lewis-Jones S. (2006). Loss-of-function mutations in the gene encoding filaggrin cause ichthyosis vulgaris. Nat. Genet..

[68] Irvine A.D., McLean W.H. (2006). Breaking the (un)sound barrier: Filaggrin is a major gene for atopic dermatitis. J. Investig. Dermatol..

[69] Presland R.B., Coulombe P.A., Eckert R.L., Mao-Qiang M., Feingold K.R., Elias P.M. (2004). Barrier function in transgenic mice overexpressing K16, involucrin, and filaggrin in the suprabasal epidermis. J. Investig. Dermatol..

[70] Bragulla H.H., Homberger D.G. (2009). Structure and functions of keratin proteins in simple, stratified, keratinized and cornified epithelia. J. Anat..

[71] Hou Y., Yao K., Yin Y., Wu G. (2016). Endogenous synthesis of amino acids limits growth, lactation, and reproduction in animals. Adv. Nutr..

[72] Zhao R., Bruning E., Rossetti D., Starcher B., Seiberg M., Iotsova-Stone V. (2009). Extracts from Glycine max (soybean) induce elastin synthesis and inhibit elastase activity. Exp. Dermatol..

[73] Karna E., Szoka L., Huynh T.Y.L., Palka J.A. (2020). Proline-dependent regulation of collagen metabolism. Cell Mol. Life Sci..

[74] Rauscher S., Baud S., Miao M., Keeley F.W., Pomès R. (2006). Proline and glycine control protein self-organization into elastomeric or amyloid fibrils. Structure.

[75] Saibi W., Feki K., Yacoubi I., Brini F. (2015). Bridging between proline structure, functions, metabolism, and involvement in organism physiology. Appl. Biochem. Biotechnol..

[76] Gorres K.L., Raines R.T. (2010). Prolyl 4-hydroxylase. Crit. Rev. Biochem. Mol. Biol..

[77] Wu Z., Hou Y., Dai Z., Hu C.A., Wu G. (2019). Metabolism, nutrition, and redox signaling of hydroxyproline. Antioxid. Redox Signal..

[78] Aoki M., Suto K., Komatsu M., Kamimura A., Morishita K., Yamasaki M., Takao T. (2012). Increasing effect of an oral intake of L-hydroxyproline on the soluble collagen content of skin and collagen fragments in rat serum. Biosci. Biotechnol. Biochem..

[79] Awuchi C.G., Igwe V.S., Amagwula I.O., Echeta C.K. (2020). Health Benefits of Micronutrients (Vitamins and Minerals) and their Associated Deficiency Diseases: A Systematic Review. Int. J. Food Sci..

[80] Dyer D.G., Dunn J.A., Thorpe S.R., Bailie K.E., Lyons T.J., McCance D.R., Baynes J.W. (1993). Accumulation of Maillard reaction products in skin collagen in diabetes and aging. J. Clin. Investig..

[81] Wu G., Fang Y.Z., Yang S., Lupton J.R., Turner N.D. (2004). Glutathione metabolism and its implications for health. J. Nutr..

[82] Imokawa G. (1989). Analysis of initial melanogenesis including tyrosinase transfer and melanosome differentiation through interrupted melanization by glutathione. J. Investig. Dermatol..

[83] del Marmol V., Solano F., Sels A., Huez G., Libert A., Lejeune F., Ghanem G. (1993). Glutathione depletion increases tyrosinase activity in human melanoma cells. J. Investig. Dermatol..

[84] del Marmol V., Ito S., Bouchard B., Libert A., Wakamatsu K., Ghanem G., Solano F. (1996). Cysteine deprivation promotes eumelanogenesis in human melanoma cells. J. Investig. Dermatol..

[85] Wu G., Morris Jr S.M. (1998). Arginine metabolism: Nitric oxide and beyond. Biochem. J..

[86] Wu G., Bazer F.W., Davis T.A., Kim S.W., Li P., Marc Rhoads J., Carey Satterfield M., Smith S.B., Spencer T.E., Yin Y. (2009). Arginine metabolism and nutrition in growth, health and disease. Amino Acids.

[87] Stechmiller J.K., Childress B., Cowan L. (2005). Arginine supplementation and wound healing. Nutr. Clin. Pract..

[88] Shindo Y., Witt E., Han D., Packer L. (1994). Dose-response effects of acute ultraviolet irradiation on antioxidants and molecular markers of oxidation in murine epidermis and dermis. J. Investig. Dermatol..

[89] Rhie G., Shin M.H., Seo J.Y., Choi W.W., Cho K.H., Kim K.H., Park K.C., Eun H.C., Chung J.H. (2001). Aging- and photoaging-dependent changes of enzymic and nonenzymic antioxidants in the epidermis and dermis of human skin in vivo. J. Investig. Dermatol..

[90] Shindo Y., Witt E., Han D., Epstein W., Packer L. (1994). Enzymic and non-enzymic antioxidants in epidermis and dermis of human skin. J. Investig. Dermatol..

[91] Shindo Y., Witt E., Packer L. (1993). Antioxidant defense mechanisms in murine epidermis and dermis and their responses to ultraviolet light. J. Investig. Dermatol..

[92] Weber S.U., Thiele J.J., Cross C.E., Packer L. (1999). Vitamin C, uric acid, and glutathione gradients in murine stratum corneum and their susceptibility to ozone exposure. J. Investig. Dermatol..

[93] Hinek A., Kim H.J., Wang Y., Wang A., Mitts T.F. (2014). Sodium L-ascorbate enhances elastic fibers deposition by fibroblasts from normal and pathologic human skin. J. Dermatol. Sci..

[94] Ivanov V., Ivanova S., Kalinovsky T., Niedzwiecki A., Rath M. (2016). Inhibition of collagen synthesis by select calcium and sodium channel blockers can be mitigated by ascorbic acid and ascorbyl palmitate. Am. J. Cardiovasc. Dis..

[95] Pasonen-Seppänen S., Suhonen T.M., Kirjavainen M., Suihko E., Urtti A., Miettinen M., Hyttinen M., Tammi M., Tammi R. (2001). Vitamin C enhances differentiation of a continuous keratinocyte cell line (REK) into epidermis with normal stratum corneum ultrastructure and functional permeability barrier. Histochem. Cell Biol..

[96] Takahashi Y., Takahashi S., Shiga Y., Yoshimi T., Miura T. (2000). Hypoxic induction of prolyl 4-hydroxylase alpha (I) in cultured cells. J. Biol. Chem..

[97] Kishimoto Y., Saito N., Kurita K., Shimokado K., Maruyama N., Ishigami A. (2013). Ascorbic acid enhances the expression of type 1 and type 4 collagen and SVCT2 in cultured human skin fibroblasts. Biochem. Biophys. Res. Commun..

[98] Ellinger S., Stehle P. (2009). Efficacy of vitamin supplementation in situations with wound healing disorders: Results from clinical intervention studie. Curr. Opin. Clin. Nutr. Metab. Care.

[99] Talarico V., Aloe M., Barreca M., Galati M.C., Raiola G. (2014). Do you remember scurvy?. Clin. Ter..

[100] Rinnerthaler M., Bischof J., Streubel M.K., Trost A., Richter K. (2015). Oxidative stress in aging human skin. Biomolecules.

[101] Kechichian E., Ezzedine K. (2018). Vitamin D and the skin: An update for dermatologists. Am. J. Clin. Dermatol..

[102] Powers H.J. (2003). Riboflavin (vitamin B-2) and health. Am. J. Clin. Nutr..

[103] Sriram K., Manzanares W., Joseph K. (2012). Thiamine in nutrition therapy. Nutr. Clin. Pract..

[104] Stover P.J., Field M.S. (2015). Vitamin B-6. Adv. Nutr..

[105] Hegyi J., Schwartz R.A., Hegyi V. (2004). Pellagra: Dermatitis, dementia, and diarrhea. Int. J. Dermatol..

[106] Spinneker A., Sola R., Lemmen V., Castillo M.J., Pietrzik K., González-Gross M. (2007). Vitamin B6 status, deficiency and its consequences—An overview. Nutr. Hosp..

[107] Daft F.S., Ashburn L.L., Sebrell W.H. (1942). Biotin deficiency and other changes in rats given sulfanilylguanidine or succinyl sulfathiazole in purified diets. Science.

[108] Dickinson A., Shao A., Boyon N., Franco J.C. (2011). Use of dietary supplements by cardiologists, dermatologists and orthopedists: Report of a survey. Nutr. J..

[109] Aaron S., Kumar S., Vijayan J., Jacob J., Alexander M., Gnanamuthu C. (2005). Clinical and laboratory features and response to treatment in patients presenting with vitamin B12 deficiency-related neurological syndromes. Neurol. India.

[110] Graells J., Ojeda R.M., Muniesa C., Gonzalez J., Saavedra J. (2009). Glossitis with linear lesions: An early sign of vitamin B12 deficiency. J. Am. Acad. Dermatol..

[111] Poljšak B., Dahmane R.G., Godić A. (2012). Intrinsic skin aging: The role of oxidative stress. Acta Dermatovenerol. Alp. Pannonica Adriat..

[112] Khadangi F., Azzi A. (2019). Vitamin E—The Next 100 Years. IUBMB Life.

[113] Mustacich D.J., Bruno R.S., Traber M.G. (2007). Vitamin E. Vitam Horm..

[114] Lee G.Y., Han S.N. (2018). The role of vitamin E in immunity. Nutrients.

[115] Manela-Azulay M., Bagatin E. (2009). *Cosmeceuticals* *vitamins*. Clin. Dermatol..

[116] Keen M.A., Hassan I. (2016). Vitamin E in dermatology. Indian. Dermatol. Online J..

[117] Park K. (2015). Role of micronutrients in skin health and function. Biomol. Ther..

[118] Nouveau-Richard S., Yang Z., Mac-Mary S., Li L., Bastien P., Tardy I., Bouillon C., Humbert P., de Lacharrière O. (2005). Skin ageing: A comparison between Chinese and European populations. A pilot study. J. Dermatol. Sci..

[119] Gao Y., Tan J., Sang Y., Tang J., Cai X., Xue H. (2023). Preparation, structure, and biological activities of the polysaccharides from fruits and vegetables: A review. Food Biosci..

[120] Hoang H.T., Moon J.Y., Lee Y.C. (2021). Natural antioxidants from plant extracts in skincare cosmetics: Recent applications, challenges and perspectives. Cosmetics.

[121] Sławińska N., Olas B. (2022). Selected seeds as sources of bioactive compounds with/diverse biological activities. Nutrients.

[122] Dreher M.L., Davenport A.J. (2013). Hass avocado composition and potential health effects. Crit. Rev. Food Sci. Nutr..

[123] Zielinska A., Nowak I. (2017). Abundance of active ingredients in sea-buckthorn oil. Lipids Health Dis..

[124] Knaggs H., Lephart E.D. (2023). Enhancing skin anti-aging through healthy lifestyle factors. Cosmetics.

[125] Tureck C., Barboza B.P., Bricarello L.P., Retondario A., Alves M.d.A., Souza A.d.M., Fernandes R., Vasconcelos F.d.A.G.d. (2022). Scientific evidence of the association between oral intake of OMEGA-3 and OMEGA-6 fatty acids and the metabolicsyndrome in adolescents: A systematic review. Nutr. Metab. Cardiovasc. Dis..

[126] Sorokin A.V., Arnardottir H., Svirydava M., Ng Q., Baumer Y., Berg A., Pantoja C.J., Florida E.M., Teague H.L., Yang Z.H. (2023). Comparison of the dietary omega-3 fatty acids impact on murine psoriasis-like skin inflammation and associated lipid dysfunction. J. Nutr. Biochem..

[127] Dhaka V., Gulia N., Ahlawat K.S., Khatkar B.S. (2011). Trans fats-sources, health risks and alternative approach—A review. J. Food Sci. Technol..

[128] Britten-Jones A.C., Craig J.P., Downie L.E. (2023). Omega-3 polyunsaturated fatty acids and corneal nerve health: Current evidence and future directions. Ocul. Surf..

[129] Opálka L., Meyer J.M., Ondrejčeková V., Svatošová L., Radner F.P.W., Vávrová K. (2022). ω-O-Acylceramides but not ω-hydroxy ceramides are required for healthy lamellar phase architecture of skin barrier lipids. J Lipid Res..

[130] Priyadarshini Pradhan S., Padhi S., Dash M., Heena Mittu B., Kour J., Nayik G.A. (2022). Behera A: Chapter 7—Carotenoids. Nutraceuticals and Health Care.

[131] Elvira-Torales L.I., García-Alonso J., Periago-Caston M.J. (2019). Nutritional importance/of carotenoids and their effect on liver health: A Review. Antioxidants.

[132] Maoka T. (2020). Carotenoids as natural functional pigments. J. Nat. Med..

[133] Zerres S., Stahl W. (2020). Carotenoids in human skin. Biochim. Biophys. Acta Mol. Cell Biol. Lipids.

[134] Rawlings A.V. (2006). Ethnic skin types: Are there differences in skin structure and function?. Int. J. Cosmet. Sci..

[135] Zouboulis C.C., Makrantonaki E., Nikolakis G. (2019). When the skin is in the center of interest: An aging issue. Clin. Dermatol..

[136] Kligman A.M. (1979). Perspectives and problems in cutaneous gerontology. J. Investig. Dermatol..

[137] Kurban R.S., Kurban A.K. (1993). Common skin disorders of aging: Diagnosis and treatment. Geriatrics.

[138] Blume-Peytavi U., Kottner J., Sterry W., Hodin M.W., Griffiths T.W., Watson R.E., Hay R.J., Griffiths C.E. (2016). Age-associated skin conditions and diseases: Current perspectives and future options. Gerontologist.

[139] Choi E.H., Man M.Q., Xu P., Xin S., Liu Z., Crumrine D.A., Jiang Y.J., Fluhr J.W., Feingold K.R., Elias P.M. (2007). Stratum corneum acidification is impaired in moderately aged human and murine skin. J. Investig. Dermatol..

[140] López-Otín C., Blasco M.A., Partridge L., Serrano M., Kroemer G. (2013). The hallmarks of aging. Cell.

[141] Makrantonaki E., Zouboulis C.C., William J. (2007). Cunliffe scientific awards. characteristics and pathomechanisms of endogenously aged skin. Dermatology.

[142] Lovell C.R., Smolenski K.A., Duance V.C., Light N.D., Young S., Dyson M. (1987). Type I and III collagen content and fibre distribution in normal human skin during ageing. Br. J. Dermatol..

[143] Autio P., Risteli J., Haukipuro K., Risteli L., Oikarinen A. (1994). Collagen synthesis in human skin in vivo: Modulation by aging, ultraviolet B irradiation and localization. Photodermatol. Photoimmunol. Photomed..

[144] Braverman I.M., Fonferko E. (1982). Studies in cutaneous aging: I. The elastic fiber network. J. Investig. Dermatol..

[145] Wang A.S., Dreesen O. (2018). Biomarkers of cellular senescence and skin aging. Front. Genet..

[146] Bentov I., Reed M.J. (2015). The effect of aging on the cutaneous microvasculature. Microvasc. Res..

[147] Li W., Chi N., Rathnayake R.A.C., Wang R. (2021). Distinctive roles of fibrillar collagen I and collagen III in mediating fibroblast-matrix interaction: A nanoscopic study. Biochem. Biophys. Res. Commun..

[148] Johnson J.M., Minson C.T., Kellogg D.L. (2014). Cutaneous vasodilator and vasoconstrictor mechanisms in temperature regulation. Compr. Physiol..

[149] Rittié L., Fisher G.J. (2015). Natural and sun-induced aging of human skin. Cold Spring Harb. Perspect. Med..

[150] Kammeyer A., Luiten R.M. (2015). Oxidation events and skin aging. Ageing Res. Rev..

[151] Choi Y.J., Moon K.M., Chung K.W., Jeong J.W., Park D., Kim D.H., Yu B.P., Chung H.Y. (2016). The underlying mechanism of proinflammatory NF-κB activation by the mTORC2/Akt/IKKα pathway during skin aging. Oncotarget.

[152] Puizina-Ivić N. (2008). Skin aging. Acta Dermatovenerol. Alp. Pannonica Adriat..

[153] Li G.Z., Eller M.S., Firoozabadi R., Gilchrest B.A. (2003). Evidence that exposure of the telomere 3′ overhang sequence induces senescence. Proc. Natl. Acad. Sci. USA.

[154] Gilchrest B.A., Eller M.S., Yaar M. (2009). Telomere-mediated effects on melanogenesis and skin aging. J. Investig. Dermatol. Symp. Proc..

[155] Ganceviciene R., Liakou A.I., Theodoridis A., Makrantonaki E., Zouboulis C.C. (2012). Skin anti-aging strategies. Dermato-Endocrinology.

[156] Veret D., Brondello J.M. (2020). Senotherapy: Advances and new clinical perspectives. Med. Sci..

[157] Tanuja Y., Mishra S., Das S., Aggarwal S., Rani V. (2015). Anticedants and natural prevention of environmental toxicants induced accelerated aging of skin. Environ. Toxicol. Pharmacol..

[158] Geissler S., Textor M., Schmidt-Bleek K., Klein O., Thiele M., Ellinghaus A., Jacobi D., Ode A., Perka C., Dienelt A. (2013). In serum veritas-in serum sanitas? Cell non-autonomous aging compromises differentiation and survival of mesenchymal stromal cells via the oxidative stress pathway. Cell Death Dis..

[159] Chen J., Li Y., Zhu Q., Li T., Lu H., Wei N., Huang Y., Shi R., Ma X., Wang X. (2017). Anti-skin-aging effect of epigallocatechin gallate by regulating epidermal growth factor receptor pathway on aging mouse model induced by d-Galactose. Mech. Ageing Dev..

[160] Bavarsad Shahripour R., Harrigan M.R., Alexandrov A.V. (2014). N-acetylcysteine (NAC) in neurological disorders: Mechanisms of action and therapeutic opportunities. Brain Behav..

[161] Bjelakovic G., Nikolova D., Gluud L.L., Simonetti R.G., Gluud C. (2012). Antioxidant supplements for prevention of mortality in healthy participants and patients with various diseases. Cochrane Database Syst. Rev..

[162] Marosz A., Chlubek D. (2014). The risk of abuse of vitamin supplements. Pomeranian J. Life Sci..

[163] Bjelakovic G., Nikolova D., Gluud C. (2014). Antioxidant supplements and mortality. Curr. Opin. Clin. Nutr. Metab. Care.

[164] Lu L.Y., Ou N., Lu Q.B. (2013). Antioxidant induces DNA damage, cell death and mutagenicity in human lung and skin normal cells. Sci. Rep..

[165] Rhee S.G. (2006). Cell signaling. H2O2, a necessary evil for cell signaling. Science.

[166] Mojallal A., Lequeux C., Shipkov C., Breton P., Foyatier J.L., Braye F., Damour O. (2009). Improvement of skin quality after fat grafting: Clinical observation and an animal study. Plast. Reconstr. Surg..

[167] Kim W.S., Park B.S., Kim H.K., Park J.S., Kim K.J., Choi J.S., Chung S.J., Kim D.D., Sung J.H. (2008). Evidence supporting antioxidant action of adipose-derived stem cells: Protection of human dermal fibroblasts from oxidative stress. J. Dermatol. Sci..

[168] Zhang S., Dong Z., Peng Z., Lu F. (2014). Anti-aging effect of adipose-derived stem cells in a mouse model of skin aging induced by D-galactose. PLoS ONE.

[169] Bernardini F.P., Gennai A., Izzo L., Zambelli A., Repaci E., Baldelli I., Fraternali-Orcioni G., Hartstein M.E., Santi P.L., Quarto R. (2015). Superficial enhanced fluid fat injection (SEFFI) to correct volume defects and skin aging of the face and periocular region. Aesthet. Surg. J..

[170] Gennai A., Zambelli A., Repaci E., Quarto R., Baldelli I., Fraternali G., Bernardini F.P. (2017). Skin rejuvenation and volume enhancement with the micro superficial enhanced fluid fat injection (M-SEFFI) for skin aging of the periocular and perioral regions. Aesthet. Surg. J..

[171] Park B.S., Jang K.A., Sung J.H., Park J.S., Kwon Y.H., Kim K.J., Kim W.S. (2008). Adipose-derived stem cells and their secretory factors as a promising therapy for skin aging. Dermatol. Surg..

[172] Fisher G.J., Datta S.C., Talwar H.S., Wang Z.Q., Varani J., Kang S., Voorhees J.J. (1996). Molecular basis of sun-induced premature skin ageing and retinoid antagonism. Nature.

[173] Kafi R., Kwak H.S., Schumacher W.E., Cho S., Hanft V.N., Hamilton T.A., King A.L., Neal J.D., Varani J., Fisher G.J. (2007). Improvement of naturally aged skin with vitamin A (retinol). Arch. Dermatol..

[174] Verdier-Sévrain S., Bonté F. (2007). Skin hydration: A review on its molecular mechanisms. J. Cosmet. Dermatol..

[175] Ramirez R.D., Morales C.P., Herbert B.S., Rohde J.M., Passons C., Shay J.W., Wright W.E. (2001). Putative telomere-independent mechanisms of replicative aging reflect inadequate growth conditions. Genes Dev..

[176] González-Suárez E., Geserick C., Flores J.M., Blasco M.A. (2005). Antagonistic effects of telomerase on cancer and aging in K5-mTert transgenic mice. Oncogene.

[177] LePillouer-Prost A., Kerob D., Nielsen M., Taieb C., Maitrot Mantelet L. (2020). Skin and menopause: Women’s point of view. J. Eur. Acad. Dermatol. Venereol..

[178] Quan T. (2023). Molecular insights of human skin epidermal and dermal aging. J Dermatol Sci..

[179] Asavasupreechar T., Saito R., Miki Y., Edwards D.P., Boonyaratanakornkit V., Sasano H. (2020). Systemic distribution of progesterone receptor subtypes in human tissues. J. Steroid Biochem. Mol. Biol..

[180] Schmidt J.B., Lindmaier A., Spona J. (1990). Hormone receptors in pubic skin of premenopausal and postmenopausal females. Gynecol. Obstet. Investig..

[181] Pelletier G., Ren L. (2004). Localization of sex steroid receptors in human skin. Histol. Histopathol..

[182] Huang A.H., Chien A.L. (2020). Photoaging: A review of current literature. Curr. Dermatol. Rep..

[183] Tran D., Townley J.P., Barnes T.M., Greive K.A. (2014). An antiaging skin care system containing alpha hydroxy acids and vitamins improves the biomechanical parameters of facial skin. Clin. Cosmet. Investig. Dermatol..

[184] Ogluszka M., Lipinski P., Starzynski R.R. (2022). Effect of Omega-3 fatty acids on telomeres-are they the elixir of youth?. Nutrients.

[185] Danby F.W. (2010). Nutrition and aging skin: Sugar and glycation. Clin. Dermatol..

[186] Dearlove R.P., Greenspan P., Hartle D.K., Swanson R.B., Hargrove J.L. (2008). Inhibition of protein glycation by extracts of culinary herbs and spices. J. Med. Food.

[187] Buckingham E.M., Klingelhutz A.J. (2011). The role of telomeres in the ageing of human skin. Exp. Dermatol..

[188] Jacczak B., Rubis B., Toton E. (2021). Potential of naturally derived compounds in telomerase and telomere modulation in skin senescence and aging. Int. J. Mol. Sci..

[189] Cai Z., Zhang J., Li H. (2019). Selenium, aging and aging-related diseases. Aging Clin. Exp. Res..

[190] Lopez-Otin C., Blasco M.A., Partridge L., Serrano M., Kroemer G. (2023). Hallmarks of aging: An expanding universe. Cell.

[191] Vinci M.C., Costantino S., Damiano G., Rurali E., Rinaldi R., Vigorelli V., Sforza A., Carulli E., Pirola S., Mastroiacovo G. (2024). Persistent epigenetic signals propel a senescence-associated secretory phenotype and trained innate immunity in CD34(+) hematopoietic stem cells from diabetic patients. Cardiovasc. Diabetol..

[192] Gao Z., Santos R.B., Rupert J., Van Drunen R., Yu Y., EckelMahan K., Kolonin M.G. (2024). Endothelial-specific telomerase inactivation causes telomere-independent cell senescence and multi-organ dysfunction characteristic of aging. Aging Cell.

[193] Thirunavukkarasu V., Nandhini A.T., Anuradha C.V. (2004). Fructose diet-induced skin collagen abnormalities are prevented by lipoic acid. Exp. Diabesity Res..

[194] Tarwadi K.V., Agte V.V. (2011). Effect of micronutrients on methylglyoxal-mediated in vitro glycation of albumin. Biol. Trace Elem. Res..

[195] Boelsma E., van de Vijver L.P., Goldbohm R.A., Klöpping-Ketelaars I.A., Hendriks H.F., Roza L. (2003). Human skin condition and its associations with nutrient concentrations in serum and diet. Am. J. Clin. Nutr..

[196] Alqanatish J.T., Alqahtani F., Alsewairi W.M., Al-kenaizan S. (2015). Childhood scurvy: An unusual cause of refusal to walk in a child. Pediatr. Rheumatol. Online J..

[197] Trapani S., Rubino C., Indolfi G., Lionetti P. (2022). A narrative review on pediatric scurvy: The last twenty years. Nutrients.

[198] Stewart M.S., Cameron G.S., Pence B.C. (1996). Antioxidant nutrients protect against UVB-induced oxidative damage to DNA of mouse keratinocytes in culture. J. Investig. Dermatol..

[199] Placzek M., Gaube S., Kerkmann U., Gilbertz K.P., Herzinger T., Haen E., Przybilla B. (2005). Ultraviolet B-induced DNA damage in human epidermis is modified by the antioxidants ascorbic acid and D-alpha-tocopherol. J. Investig. Dermatol..

[200] Zussman J., Ahdout J., Kim J. (2010). Vitamins and photoaging: Do scientific data support their use?. J. Am. Acad. Dermatol..

[201] Langton A.K., Sherratt M.J., Griffiths C.E., Watson R.E. (2010). A new wrinkle on old skin: The role of elastic fibres in skin ageing. Int. J. Cosmet. Sci..

[202] Baumann L. (2007). Skin ageing and its treatment. J. Pathol..

[203] Kaimal S., Thappa D.M. (2010). Diet in dermatology: Revisited. Indian J. Dermatol. Venereol. Leprol..

[204] Cao C., Xiao Z., Wu Y., Ge C. (2020). Diet and skin aging-from the perspective of food nutrition. Nutrients.

[205] Strasser B., Volaklis K., Fuchs D., Burtscher M. (2018). Role of dietary protein and muscular fitness on longevity and aging. Aging Dis..

[206] Balić A., Vlašić D., Žužul K., Marinović B., Bukvić Mokos Z. (2020). Omega-3 versus omega-6 polyunsaturated fatty acids in the prevention and treatment of inflammatory skin diseases. Int. J. Mol. Sci..

[207] Rawlings A.V., Scott I.R., Harding C.R., Bowser P.A. (1994). Stratum corneum moisturization at the molecular level. J. Investig. Dermatol..

[208] Schürer N.Y., Plewig G. (1991). Elias PM Stratum corneum lipid function. Dermatologica.

[209] Reuter J., Merfort I., Schempp C.M. (2010). Botanicals in dermatology: An evidence-based review. Am. J. Clin. Dermatol..

[210] Tungmunnithum D., Thongboonyou A., Pholboon A., Yangsabai A. (2018). Flavonoids and other phenolic compounds from medicinal plants for pharmaceutical and medical aspects: An overview. Medicines.

[211] Anunciato T.P., da Rocha Filho P.A. (2012). Carotenoids and polyphenols in nutricosmetics, nutraceuticals, and cosmeceuticals. J. Cosmet. Dermatol..

[212] Im A.R., Kim Y.M., Chin Y.C., Chae S. (2017). Protective effects of compounds from *Garcinia mangostana* L. (mangosteen) against UVB damage in HaCaT cells and hairless mice. Int. J. Mol. Med..

[213] Ma R.J., Yang L., Bai X., Yuan M.Y., Wang Y.K., Xie Y., Hu J.M., Zhou J. (2019). Phenolic constituents with antioxidative, tyrosinase inhibitory and anti-aging activities from *Dendrobium loddigesii* Rolfe. Nat. Prod. Bioprospecting.

[214] Morikawa T., Nagatomo A., Kitazawa K., Muraoka O., Kikuchi T., Yamada T., Tanaka R., Ninomiya K. (2018). Collagen synthesispromoting effects of andiroba oil and its limonoid constituents in normal human dermal fibroblasts. J. Oleo Sci..

[215] Tan H., Sonam T., Shimizu K. (2017). The potential of triterpenoids from loquat leaves (*Eryobotrya japonica*) for prevention and treatment of skin disorder. Int. J. Mol. Sci..

[216] Henriet E., Jäger S., Tran C., Bastien P., Michelet J.F., Minondo A.M., Formanek F., Dalko-Csiba M., Lortat-Jacob H., Breton L. (2017). A jasmonic acid derivative improves skin healing and induces changes in proteoglycan expression and glycosaminoglycan structure. Biochim. Biophys. Acta (BBA)—Gen. Subj..

[217] Kim J.E., Jang S.G., Lee C.H., Lee J.Y., Park H., Kim J.H., Lee S., Kim S.H., Park E.Y., Lee K.W. (2019). Beneficial effects on skin health using polysaccharides from red ginseng by-product. J. Food Biochem..

[218] Zdunska-Peciak K., Rotsztejn H. (2020). The effectiveness of ferulic acid and microneedling in reducing signs of photoaging: A split-face comparative study. Dermatol. Ther..

[219] Moreira L.C., Ávila R.I., Veloso D.F.M.C., Pedrosa T.N., Lima E.S., Couto R.O., Lima E.M., Batista A.C., Paula J.R., Valadares M.C. (2017). In vitro safety and efficacy of a complex botanical mixture of *Eugenia dysenterica* Dc. (Myrtaceae): Prospects for developing a new dermocosmetic product. Toxicol. In Vitr..

[220] Song E., Chung H., Shin E., Jeong J.K., Han B.K., Choi H.J., Hwang J. (2016). *Gastrodea elata* Blume extract modulates antioxidant activity and ultraviolet A-irradiated skin aging in human dermal fibroblast cells. J. Med. Food.

[221] Lourith N., Kanlayavattanakul M., Chaikul P., Chansriniyom C., Bunwatcharaphansakun P. (2017). In vitro and cellular activities of the selected fruit residues for skin aging treatment. An. Acad. Bras. Ciências.

[222] Wu L., Chen C., Cheng C., Dai H., Ai Y., Lin C., Chung Y. (2018). Evaluation of tyronisase inhibitory, antioxidant, antimicrobial, and antiaging activities of *Magnolia officinalis* extracts after *Aspergillus niger* fermentation. BioMed Res. Int..

[223] Bose B., Choudhury H., Tandon P., Kumaria S. (2017). Studies on secondary metabolite profiling, anti-inflammatory potential, in vitro photoprotective and skin-aging related enzyme inhibitory activities of *Malaxis acuminate*, a threatened orchid of nutraceutical importance. J. Photochem. Photobiol. B.

[224] Pientaweeratch S., Panapisal V., Tansirikongkol A. (2016). Antioxidant, anti-collagenase and anti-elastase activities of *Phyllanthus emblica*, *Manilkara zapota* and sylimarin: An in vitro comparative study for anti-aging applications. Pharm. Biol..

[225] Shoko T., Naharaj V.J., Naidoo D., Tselanyane M., Nthambeleni R., Khorombi E., Apostolides Z. (2018). Anti-aging potential of extracts from *Sclerocarya birrea* (A. Rich.) Hochst and its chemical profiling by UPLC-Q-TOF-MS. BMC Complement. Altern. Med..

[226] Kwon K.R., Alam M.B., Park J.H., Kim T.H., Lee S.H. (2019). Attenuation of UVB-induced photo-aging by polyphenolic-rich *Spatholobus suberectus* stem extract via modulation of MAPK/AP-1/MMPs signaling in human keratinocytes. Nutrients.

[227] Sundaran I.K., Sarangi D.D., Sundararajan V., George S., Mohideen S.S. (2018). Poly herbal formulation with anti-elastase and antioxidant properties for skin anti-aging. BMC Complement. Altern. Med..

[228] Lee H., Hong Y., Kwon S.H., Park J., Park J. (2016). Anti-aging effects of *Piper cambodianum* P. Fourn. extract on normal human dermal fibroblast cells and a wound-healing model in mice. Clin. Interv. Aging.

[229] Dieament G., Pereda M.D.C.V., Nogueira C., Eberlin S., Facchini G., Checon J.T., Cesar C.K., Mussi L., Polezel M.A., Martins-Oliveira D. (2015). Antiageing mechanisms of a standardized supercritical CO2 preparation of Black Jack (*Bidens Pilosa* L.) in human fibroblasts and skin fragments. Altern. Med..

[230] Hwang E., Ngo H.T.T., Seo S.A., Park B., Zhang M., Yi T.H. (2018). Protective effect of dietary *Alchemilla mollis* on UVB-irradiated premature skin aging through regulation of transcription factor NFATc1 and Nrf2/ARE pathways. Phytomedicine.

[231] Ngo H.T.T., Hwang E., Seo S.A., Park B., Sun Z.W., Zhang M., Shin Y.K., Yi T.H. (2017). Topical application of neem leaves prevents wrinkles formation in UVB-exposed hairless mice. J. Photochem. Photobiol. B.

[232] Zhao P., Alam M.B., Lee S.H. (2019). Protection of UVB-induced photoaging by Fuzhuan-brick tea aqueous extract via MAPKs/Nrf2-mediated down-regulation of MMP-1. Nutrients.

[233] Adhikari D., Panthi V.K., Pangeni R., Kim H.J., Park J.W. (2017). Preparation, characterization, and biological activities of topical anti-aging ingredients in a *Citrus junos* callus extract. Molecules.

[234] Nam G.H., Kawk H.W., Kim S.Y., Kim Y.M. (2020). Solvent fraction of fermented *Trapa japonica* fruit extract stimulates collagen synthesis through TGF-β1/GSK-3β/β-catenin pathway in human dermal fibroblasts. J. Cosmet. Dermatol..

[235] Jeon H., Kim D.H., Nho Y.H., Park J.E., Kim S.N., Choi E.H. (2016). A mixture of *Kochia scoparia* and *Rosa multiflora* with PPAR α/γ dual agonistic effects prevents photoaging in hairless mice. Int. J. Mol. Sci..

[236] You J., Roh K.B., Li Z., Liu G., Tang J., Shin S., Park D., Jung E. (2015). The antiaging properties of *Andrographs paniculata* by activation epidermal cell stemness. Molecules.

[237] Limtrakul P., Yodkeeree S., Thippraphan P., Punfa W., Srisomboom J. (2016). Anti-aging and tyrosinase inhibition effects of *Cassia fistula* flower butanolic extract. BMC Complement. Altern. Med..

[238] Pakravan N., Mahmoudi E., Hashemi S.A., Kamali J., Hajiaghayi R., Rahimzadeh M. (2017). Cosmeceutical effect of ethyl acetate fraction of kombucha tea by intradermal administration in the skin of aged mice. J. Cosmet. Dermatol..

[239] Bravo K., Duque L., Ferreres F., Moreno D.A., Osorio E. (2017). *Passiflora tarminiana* fruits reduce UVB-induced photoaging in human skin fibroblasts. J. Photochem. Photobiol. B.

[240] Cicchetti E., Duroure L., Le Borgne E., Laville R. (2018). Upregulation of skin-aging biomarkers in aged NHDF cells by a sucrose ester extract from agroindustrial waste of *Physalis peruviana* calyces. J. Nat. Prod..

[241] Jeong D., Lee J., Park S.H., Kim Y.A., Park B.J., Oh J., Sung G.H., Aravithan A., Kim J.H., Kang H. (2019). Antiphotoaging and antimelanogenic effects of *Penthorum chinense* pursh ethanol extract due to antioxidant- and autophagy-inducing properties. Oxidative Med. Cell. Longev..

[242] Khare R., Upmanyu N., Jha M. (2021). Exploring the potential of methanolic extract of *Salvia officinalis* against UV exposed skin aging: In vivo and in vitro model. Curr. Aging Sci..

[243] Kim H.K. (2016). Protective effect of garlic on cellular senescence in UVB-exposed HaCaT human keratinocytes. Nutrient.

[244] Hwang E., Lin P., Ngo T.T., Yi T.H. (2018). Clove attenuates UVB-induced photodamage and repairs skin barrier function in hairless mice. Food Nutr..

[245] Choi S.I., Lee J.S., Lee S., Cho B.Y., Choi S.H., Han X., Sin W.S., Kim Y.C., Lee B.Y., Kang I.J. (2019). Protective effects and mechanisms of *Pourthiaea villosa* (Thumb.) Decne. extract on hydrogen peroxide-induced skin aging in human dermal fibroblasts. J. Med. Food.

[246] Kusumawati I., Kurniawan K.O., Rullyansyah S., Prijo T.A., Widyowati R., Ekowati J., Hestianah E.P., Maat S., Matsunami K. (2018). Anti-aging of *Curcuma heyneana* Valeton & Zipj: A scientific approach use in Javanese tradition. J. Ethnopharmacol..

[247] Li J., Lu Y.R., Lin I.F., Kang W., Chen H.B., Lu H.F., Wang H.M.D. (2020). Reversing UVB-induced photoaging with *Hibiscus sabdariffacalyx* aqueous extract. J. Sci. Food Agric..

[248] Choi M., Oh J.H., Shin M.K., Lee S.R. (2015). Beneficial effects of blood group antigen synthesis-increasing natural plant extracts and monosaccharides on extracellular matrix protein production in vivo. J. Dermatol. Sci..

[249] Uchiyama T., Tsunenaga M., Miyanaga M., Ueda U., Ogo M. (2018). Oral intake of lingonberry and amla fruit extract improves skin conditions in healthy female subjects: A randomized, double-blind placebo-controlled clinical trial. Biotechnol. Appl. Biochem..

[250] Caversan J., Mussi L., Sufi B., Padovani G., Nazaro L., Camargo-Junior F.B., Magalhães W.V., Di Stasi L.C. (2021). A new phytocosmetic preparation from *Thymus vulgaris* stimulates adipogenesis and controls skin aging process: In vitro studies and topical effects in a double-blind placebo-controlled clinical trial. J. Cosmet. Dermatol..

